# Integrative analysis of the microRNA-mRNA response to radiochemotherapy in primary head and neck squamous cell carcinoma cells

**DOI:** 10.1186/s12864-015-1865-x

**Published:** 2015-09-02

**Authors:** Isolde Summerer, Julia Hess, Adriana Pitea, Kristian Unger, Ludwig Hieber, Martin Selmansberger, Kirsten Lauber, Horst Zitzelsberger

**Affiliations:** Research Unit Radiation Cytogenetics, Helmholtz Center Munich, Ingolstaedter Landstr.1, 85764 Neuherberg, Germany; Clinical Cooperation Group ‘Personalized Radiotherapy of Head and Neck Cancer’, Helmholtz Center Munich, Ingolstaedter Landstr. 1, 85764 Neuherberg, Germany; Department of Radiation Oncology, University of Munich, Marchioninistr. 15, 81377 Munich, Germany

**Keywords:** Pathway enrichment analysis, Interaction network, Head and neck squamous cell carcinoma, Integrative biology, Radiochemotherapy, microRNA, HNSCC cell culture model

## Abstract

**Background:**

Head and neck squamous cell carcinoma (HNSCC) is a very heterogeneous disease resulting in huge differences in the treatment response. New individualized therapy strategies including molecular targeting might help to improve treatment success. In order to identify potential targets, we developed a HNSCC radiochemotherapy cell culture model of primary HNSCC cells derived from two different patients (HN1957 and HN2092) and applied an integrative microRNA (miRNA) and mRNA analysis in order to gain information on the biological networks and processes of the cellular therapy response. We further identified potential target genes of four therapy-responsive miRNAs detected previously in the circulation of HNSCC patients by pathway enrichment analysis.

**Results:**

The two primary cell cultures differ in global copy number alterations and *P53* mutational status, thus reflecting heterogeneity of HNSCC. However, they also share many copy number alterations and chromosomal rearrangements as well as deregulated therapy-responsive miRNAs and mRNAs. Accordingly, six common therapy-responsive pathways (*direct P53 effectors*, *apoptotic execution phase*, *DNA damage/telomere stress induced senescence*, *cholesterol biosynthesis*, *unfolded protein response*, *dissolution of fibrin clot*) were identified in both cell cultures based on deregulated mRNAs. However, inflammatory pathways represented an important part of the treatment response only in HN1957, pointing to differences in the treatment responses of the two primary cultures. Focused analysis of target genes of four therapy-responsive circulating miRNAs, identified in a previous study on HNSCC patients, revealed a major impact on the pathways *direct P53 effectors*, the *E2F transcription factor network* and *pathways in cancer* (mainly represented by the *PTEN/AKT* signaling pathway).

**Conclusions:**

The integrative analysis combining miRNA expression, mRNA expression and the related cellular pathways revealed that the majority of radiochemotherapy-responsive pathways in primary HNSCC cells are related to cell cycle, proliferation, cell death and stress response (including inflammation). Despite the heterogeneity of HNSCC, the two primary cell cultures exhibited strong similarities in the treatment response. The findings of our study suggest potential therapeutic targets in the *E2F transcription factor network* and the *PTEN/AKT* signaling pathway.

**Electronic supplementary material:**

The online version of this article (doi:10.1186/s12864-015-1865-x) contains supplementary material, which is available to authorized users.

## Background

Head and neck squamous cell carcinoma (HNSCC) includes epithelial cancers of the lip, oral cavity, nasal cavity, paranasal sinuses, salivary glands, larynx and pharynx (nasopharynx, oropharynx and hypopharynx) [1] and represents the sixth most common cancer in the world [2] with an average 5-year survival rate of approximately 65 % [3]. Some of the tumors are unresectable because of their complex anatomy [4]. In addition, HNSCC is usually not detected in the early stages of the disease due to the lack of clinical symptoms, which aggravates treatment [5]. The challenges in treating HNSCC tumors are functional preservation of substantial organs, such as salivary glands, and minimization of side effects, such as dysphagia. Moreover, HNSCC tumors show a high degree of heterogeneity and variation in the therapeutic response requiring individualized treatment strategies [6, 7]. In order to address these issues, combined and targeted treatment strategies as well as more effective treatment monitoring is needed to improve therapy outcomes and patients’ quality of life.

MicroRNAs (miRNAs) represent a class of non-coding RNAs acting as posttranscriptional gene expression regulators by inhibiting translation or destabilizing mRNAs. They are known to be involved in regulating and coordinating multiple cellular pathways and processes. MiRNAs show a response to various cellular stressors and are key players in many diseases such as cancer [8]. Specific miRNA signatures were discovered for several tumor types [9]. For HNSCC a considerable number of miRNAs were identified as promising molecular biomarkers for diagnosis and prognosis targeting either oncogenic or tumor suppressor transcripts [10–12]. However, there is still uncertainty concerning the functional role of most of the miRNAs since one miRNA may target multiple mRNAs while one mRNA can be regulated by a number of different miRNAs.

Network-based integrative analysis combining molecular data from multiple levels represents a valuable tool for a better understanding of complex signaling networks and related biological processes. Correlation analysis of expression values of potentially interacting molecules enables reconstruction of interaction networks based on experimental data. In this study integrative analysis of miRNA and mRNA profiles based on the identification of correlating expression patterns revealed potential functional relationships and pathways involved in the cellular treatment response [13]. The analysis can be strengthened by integration of data bases on previously validated target interactions. Another tool for the *in silico* investigation of interactions is pathway enrichment analysis, which annotates molecules of interest, e.g. differentially expressed genes, to cellular pathways based on over-representation using the information of pathway databases, such as Reactome [14].

The aim of the current study was to shed light on the cellular functions of therapy-responsive miRNAs and to gain additional information on the treatment effects on cellular processes and pathways in order to enable the identification of potential therapeutic targets. For this purpose we used primary HNSCC cells as a cell culture model for radiochemotherapy [15] and performed integrative analysis of the miRNA and mRNA expression profiles in order to analyze affected pathways for a better understanding of the response of HNSCC cells to radiochemotherapy.

We aimed to validate our *in vitro* data by focusing on a therapy-responsive network of patient-derived data from a previous study [15].

## Results

### Characterization of the primary HNSCC cell lines

The newly established HNSCC cell lines HN1957 (nasopharynx) and HN2092 (oral cavity) were published in a previous study, where a cell culture model was established to simulate radiochemotherapy of a HNSCC patient cohort *in vitro* [15]. For the cell culture model primary cell cultures were selected instead of established cell lines since the features of primary cells are closer to the conditions in the patient. A further selection criterion for the primary cell lines was that they were derived from tumor sites, that were also represented in the HNSCC patient cohort [15]. Apart from that, we selected one *P53*-mutated (HN1957) and one *P53* wild type (HN2092) primary cell line. A nasopharyngeal carcinoma was included since standard treatment for these tumors is radiotherapy or radiochemotherapy due to their high sensitivity towards this treatment [16]. Characteristics of the primary cells lines are listed in Table 1. In the present study we used the radiochemotherapy cell culture model in order to gain information on the molecular radiochemotherapy response. As it was already shown before, HN1957 demonstrated a higher decrease in cellular viability following treatment with ionizing radiation and 5-fluorouracil (5-FU) compared to HN2092 [15]. To further characterize the two cell lines in this study we conducted array comparative genomic hybridization CGH (array CGH) analysis, spectral karyotyping (SKY), *P53* and *EGFR* sequencing analysis as well as *EGFR* and *EpCAM* surface expression.

**Table 1:** Characteristics of primary HNSCC cell cultures

Case	HN1957	HN2092
Gender of patient	f	m
Age at diagnosis, years	85	73
Tumor site	left maxilla / left nasal floor	right floor of mouth
TNM	n.a.	pT4pN0
HPV-status	negative	negative
EBV-status	negative	n.a.
P53-status	mutated	wild type
Radiosensitivity		
α (+/-SD)	0.094 (+/− 0.022)*	0.614 (+/− 0.019)*
β (+/-SD)	0.038 (+/− 0.004)**	0.021 (+/− 0.003)**
SF2	0.71	0.27
Cell type	epithelial	epithelial

*n.a.* Not available, *SD* Standard deviation, *SF2* Surviving fraction at 2 Gy

*ttest of α values results in significant difference between HN1957 and HN2092 (*p* < 0.05)

**ttest of β values results in significant difference between HN1957 and HN2092 (*p* < 0.05)

Array CGH demonstrated 30 copy number alterations involving 18 chromosomes in HN1957 and 46 copy number alterations involving 19 chromosomes in HN2092 (Additional files 1, 2, 3A and 4A). SKY revealed the following clonal karyotype for HN1957 resulting from evaluation of 16 metaphases: 65-81,XX,+X,+del(X)(p13 →qter),+1,+2,+del(2)(p13 → qter),+3,+der(3)t(3;14)(p11 →qter;qter → q11),+4,+5,+i(5)(p10),+6,+7,+i(7)(p10),+8,+der(8)t(5;8)(?;p10 → qter),+9,+der(9)t(X;9)(?;p13 → qter),+10,+der(10)t(10;17)(p10 → qter;qter →q10),+11,+12,+13,der(14)t(13;14)(qter → q11;p11 → qter),+15,i(15)(q10),+16,+17,+19,+20,+21,+22,i(22)(q10). A representative metaphase is shown in Additional file 3B.

HN2092 exhibited the following clonal karyotype resulting from evaluation of 15 metaphases: 69-77,X,Y,+Y,+i(X)(p10),+i(X)(q10),+der(1)t(1;21)(p11 → qter;qter → q11),+2,+3,+der(4)t(1;4)(pter → q21;?),+der(4)t(1;4)(qter →q10;q10 → qter),+5,+i(5)(p10),+6,+der(7)addv(7)(q31)t(7;11)(?;?),+der(8)t(8;14)(p11 → qter;qter → q11),+der(9)t(9;13)(p11 → qter;qter → q14),+10,+11,+12,der(13)t(12;13)(?;p13 →q22),+14,der(15)t(3;15)(?;p11 → qter),+16,+17,+19,+20,+22. Additional file 4B shows a representative metaphase. The karyotypes reflected many of the copy number alterations that were detected by array CGH in HN1957 (isochromosomes, gains of chromosomes and chromosome arms: 5p, 7p, 8, 9, 11, 13q, 15, 17q, 20) and HN2092 (isochromosomes, gains of chromosomes and chromosome arms: 5p, 8, 9, 11q, 12q, 13q, 14, 16, 20).

Further, sequencing analysis revealed no mutations of *EGFR* in both cell cultures, but two point mutations of the *P53* gene in HN1957 (P72R and Δ331). Both cell cultures strongly overexpressed *EGFR* and *EpCAM* compared to OKF6-hTERT keratinocytes as determined by flow cytometry surface staining (Additional file 5).

### MiRNA and mRNA expression following radiochemotherapy treatment

**Fig. 1:**
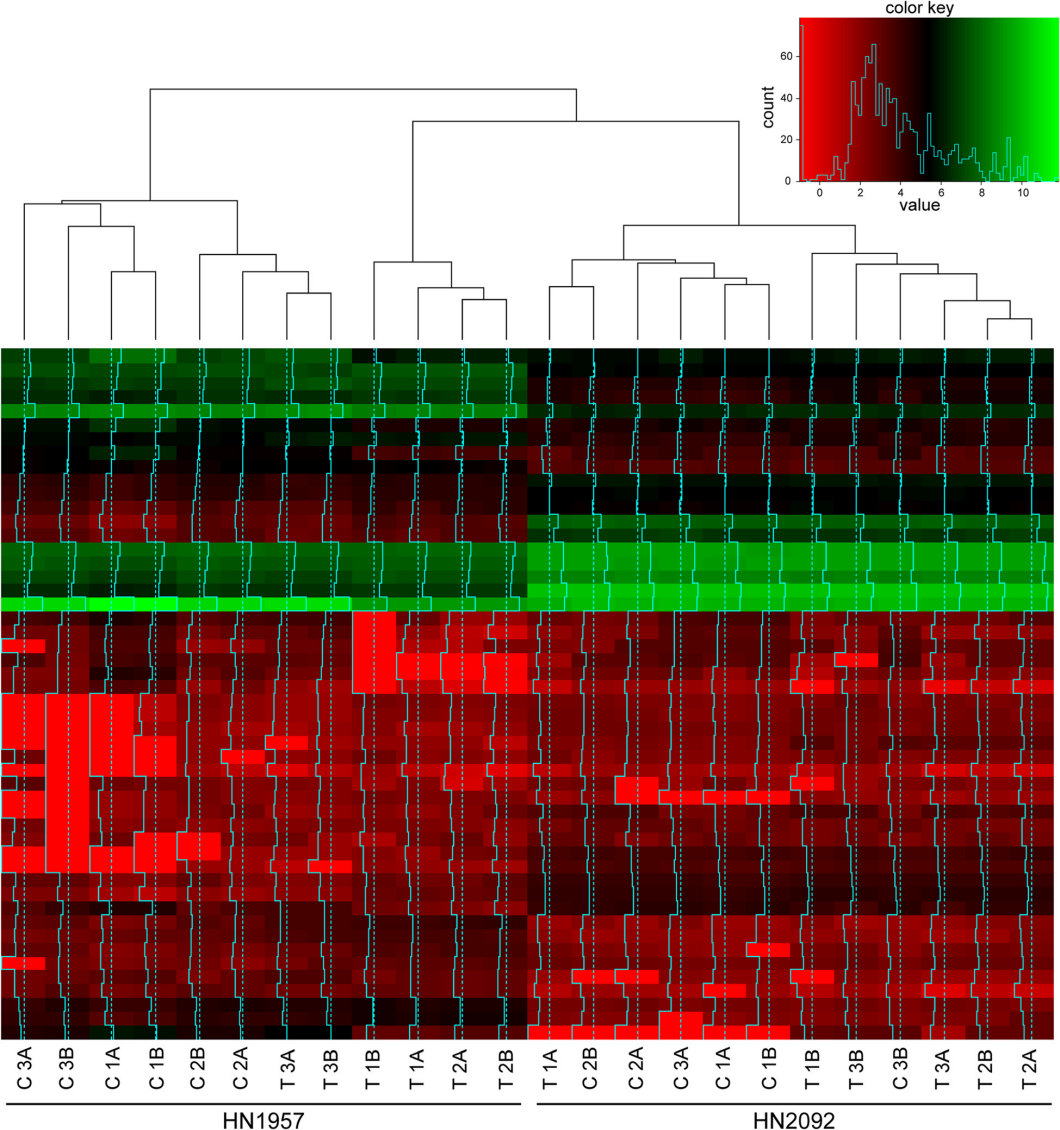
Unsupervised hierarchical cluster analysis of the expression levels of the top 50 differentially expressed miRNAs in untreated and radiochemotherapy treated HN1957 and HN2092 primary HNSCC cells. Control samples (C) were treated with DMSO and sham-irradiated, treated samples (T) were treated with 5-FU and irradiated with 2 × 2 Gy. A and B represent technical replicates; 1, 2 and 3 represent biological replicates

In order to analyze common features and differences in the radiochemotherapy response on the miRNA and mRNA level, expression changes were assessed following treatment in both primary cell lines. Significantly deregulated miRNAs in the primary cells after radiochemotherapy treatment were previously reported by Summerer et al. [15]. A heatmap of the expression profiles of the top 50 deregulated miRNAs revealed distinctive patterns for the two different tumor cell cultures (Fisher’s exact test *p* = 0.001) as well as for untreated and treated samples (three biological and two technical replicates each) resulting in significant or close to significant clustering (Fisher’s exact test HN1957: *p* = 0.015, HN2092: *p* = 0.080) (Fig. 1). In HN1957 57 significantly deregulated miRNAs were identified while HN2092 showed deregulated expression of 79 miRNAs with an overlap of 27 miRNAs between the two cell cultures.

**Fig. 2:**
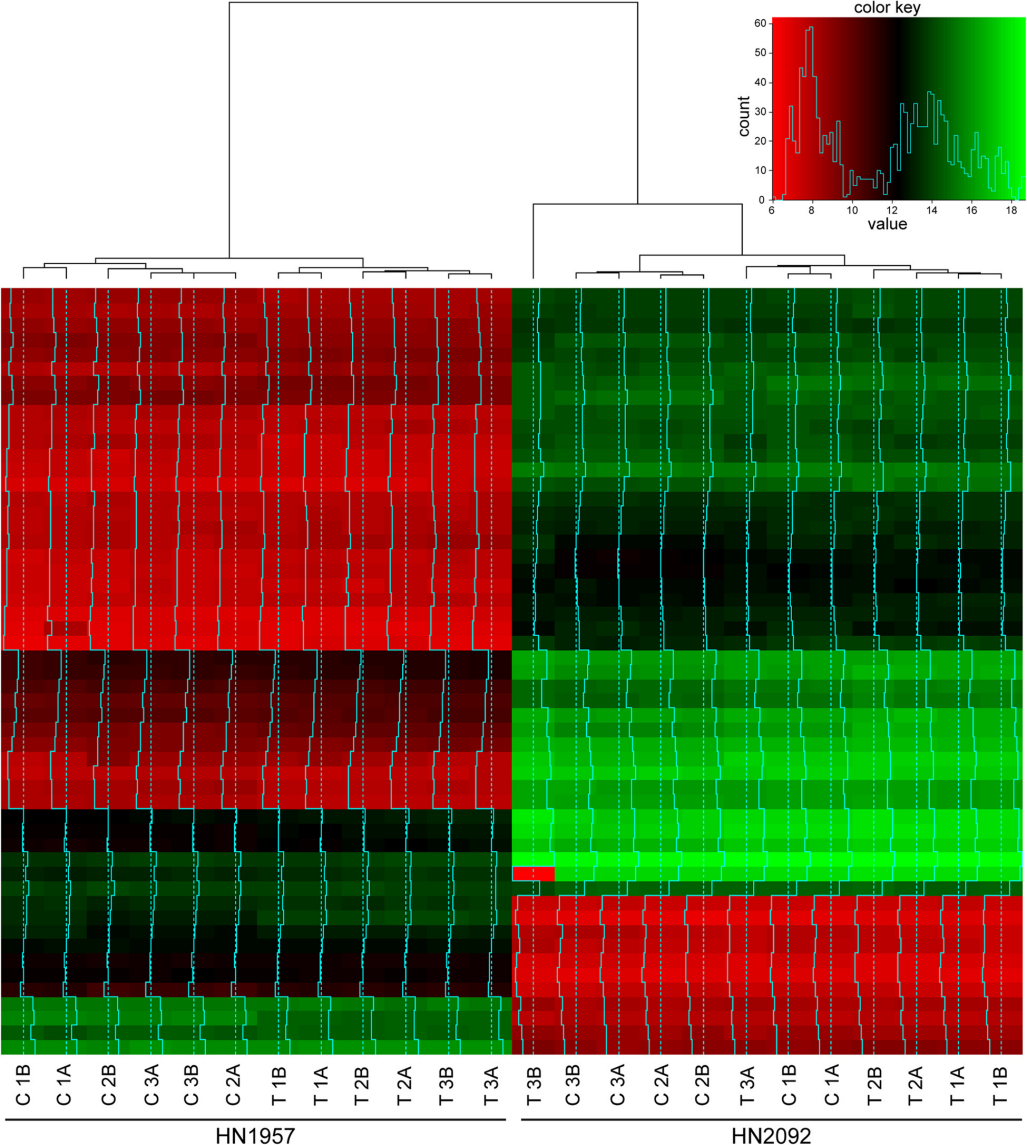
Unsupervised hierarchical cluster analysis of the expression levels of the top 50 differentially expressed mRNAs in untreated and radiochemotherapy treated HN1957 and HN2092 primary HNSCC cells. Control samples (C) were treated with DMSO and sham-irradiated, treated samples (T) were treated with 5-FU and irradiated with 2 × 2 Gy. A and B represent technical replicates; 1, 2 and 3 represent biological replicates

Global mRNA expression was measured for both cultures after radiochemotherapy or sham-treatment and unsupervised hierarchical clustering of the gene expression patterns using the 50 mRNAs with the highest variance resulted in two main clusters separating samples of the two cell cultures (Fisher’s exact test *p* = 0). Further, the cluster analysis revealed significant or border line significant separation of control samples and treated samples (Fisher’s Exact test HN1957: *p* = 0, HN2092: *p* = 0.061) (Fig. 2).

For HN1957 612 genes (Additional file 6) and for HN2092 598 genes (Additional file 7) were significantly (adjusted *p*-value < 0.05) deregulated after radiochemotherapy treatment with an overlap of 190 genes between both primary cultures.

### Pathway enrichment analysis

For a comprehensive insight in the cellular pathways, which were affected by the radiochemotherapy treatment, a pathway enrichment analysis was applied based on the significantly deregulated mRNAs. The analysis exhibited *DNA damage/telomere stress induced senescence*, *direct P53 effectors*, *cholesterol biosynthesis*, *dissolution of fibrin clot*, *unfolded protein response* and *apoptotic execution phase* as overlap of significantly (FDR < 0.05) enriched pathways (Additional files 8 and 9). Differences in the treatment response between the two primary cultures are reflected by pathways such as *TGF-beta signaling pathway*, *regulation of nuclear SMAD2/3 signaling*, *TNF signaling pathway* and *IL6-mediated signaling events*, which play a role only in the treatment response in HN1957, but not in HN2092.

### MiRNA-mRNA interactions

**Fig. 3:**
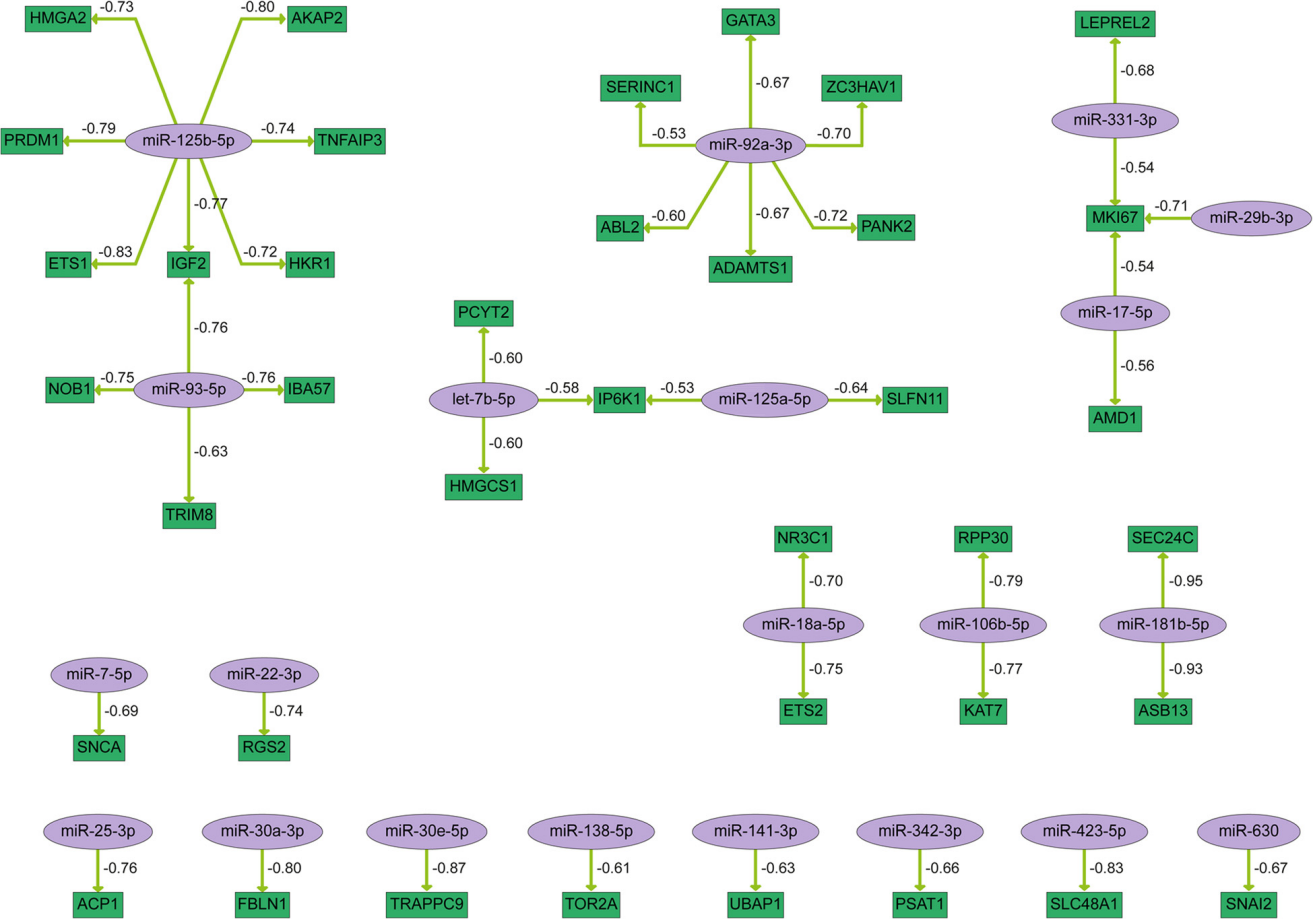
MiRNA-mRNA interaction network reflecting the response to radiochemotherapy treatment in HN1957. MiRNA-mRNA pairs were generated based on the correlation coefficient (c ≤−0.5) of their expression levels. MiRNAs are shown in purple, potential target genes are shown in green. Arrows indicate the direction of regulation. The numbers refer to the correlation value of the respective miRNA and mRNA expression levels

**Fig. 4:**
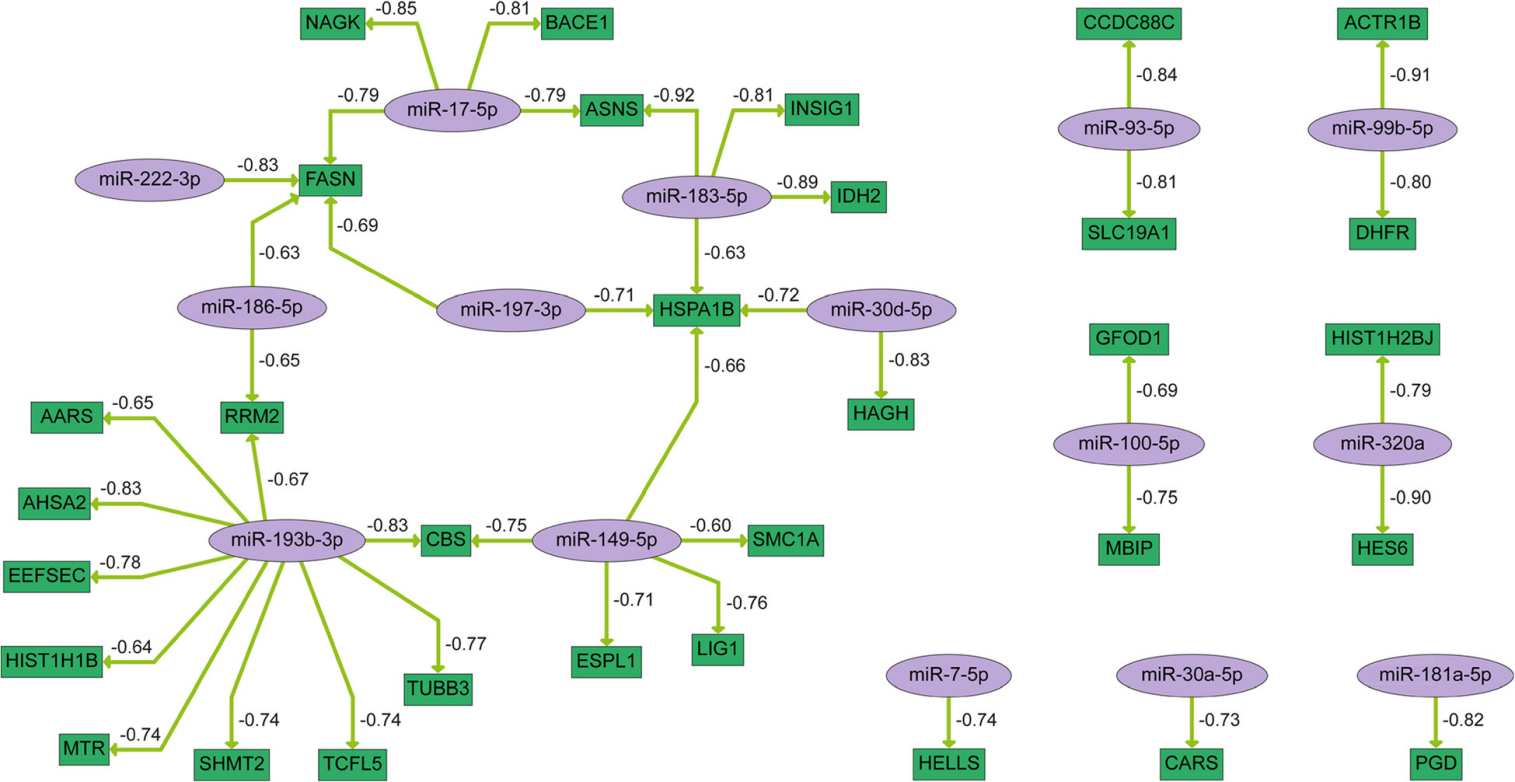
MiRNA-mRNA interaction network reflecting the response to radiochemotherapy treatment in HN2092. MiRNA-mRNA pairs were generated based on the correlation coefficient (c ≤−0.5) of their expression levels. MiRNAs are shown in purple, potential target genes are shown in green. Arrows indicate the direction of regulation. The numbers refer to the correlation value of the respective miRNA and mRNA expression levels

**Fig. 5:**
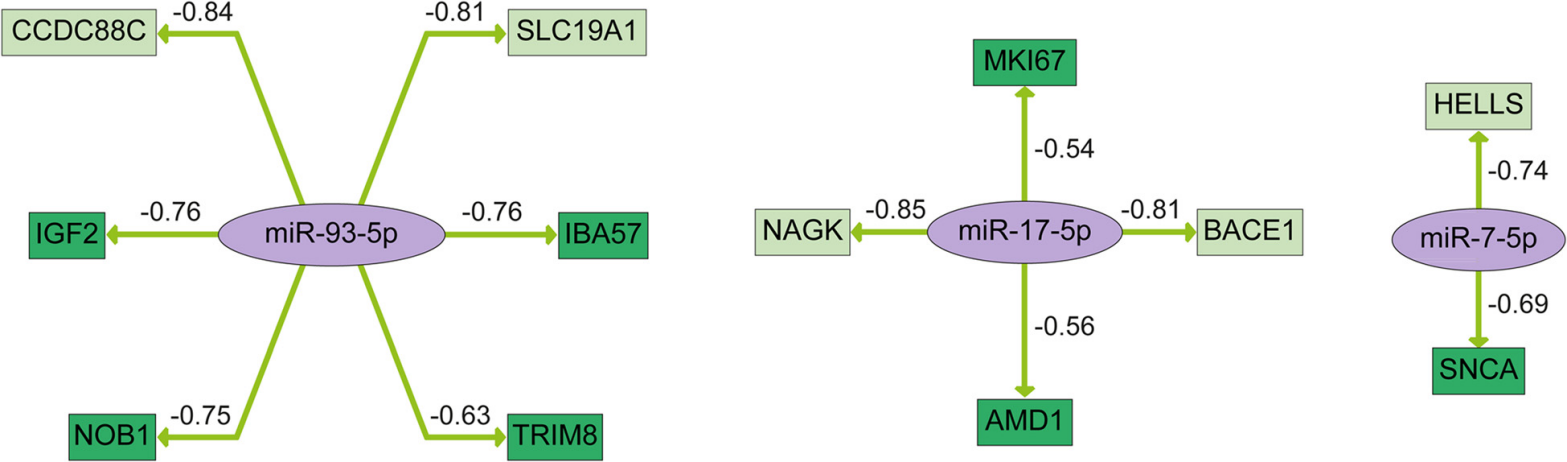
Combined network reflecting common miRNA-mRNA interactions between HN1957 and HN2092 in response to radiochemotherapy treatment. MiRNA-mRNA pairs were generated based on the correlation coefficient (c ≤−0.5) of their expression levels. MiRNAs are shown in purple, potential target genes in HN1957 are shown in dark green, potential target genes in HN2092 in light green. Arrows indicate the direction of regulation. The numbers refer to the correlation values of the respective miRNA and mRNA expression levels

We further aimed to identify potential miRNA-mRNA interactions that are part of the treatment response in order to gain information on the function of the treatment-responsive miRNAs. Integrative network analysis of significantly deregulated miRNAs and differentially expressed mRNAs including adjustment with validated miRNA-mRNA interactions derived from the miRTarBase [17, 18] resulted in functional miRNA-mRNA networks affected by radiochemotherapy treatment in HN1957 (Fig. 3) and HN2092 (Fig. 4). The miRNAs appearing in the interaction networks of both primary cell lines and their corresponding target mRNAs are combined in Fig. 5.

For technical validation by quantitative real-time PCR (qRT-PCR) individual miRNA and mRNA candidates were selected according to the following criteria: miRNA-mRNA correlation values ≤ −0.8, tumor-related genes or deregulated miRNAs in blood plasma of radiochemotherapy-treated HNSCC patients (Tables 2 and 3). In HN1957 upregulation of *miR-181b-5p* (*p* = 0.008) as well as *miR-425-5p* (with a *p*-value close to the significance level, *p* = 0.052) was confirmed. Moreover, for the target genes of *miR-181b-5p, ASB13* (*p* = 0.004) and *SEC24C* (*p* = 0.018), a significant downregulation was confirmed by qRT-PCR as well as downregulation of *TRAPPC9* (*p* = 0.046). In HN2092 downregulation of *miR-93-5p* (*p* < 0.001) as well as upregulation of *miR-181a-5p* (*p* = 0.001) was validated. *MiR-183-5p* was upregulated (with a *p*-value close to the significance level, *p* = 0.071) while downregulation of its target genes *ASNS* (*p* = 0.017) and *IDH2* (*p* = 0.045) was confirmed by qRT-PCR. Additionally, downregulation of *ACTR1B* (*p* = 0.012) and *FASN* (*p* = 0.007) was verified.

**Table 2:** Validation of deregulated miRNAs and correlating target mRNAs in HN1957 after radiochemotherapy treatment (analyzed with Agilent microarrays and TaqMan single qRT-PCR assays)

miRNA	Array	qRT-PCR	mRNA	Array	qRT-PCR
	FC (*p* value)	FC (*p* value)		FC (*p* value)	FC (*p* value)
*miR-25-3p*	0.88 (0.037)	0.89 (0.152)	*-*	*-*	*-*
*miR-30a-3p*	1.33 (0.013)	1.17 (0.294)	*FBLN1*	0.68 (<0.001)	0.98 (0.775)
*miR-30e-5p*	1.23 (0.010)	0.83 (0.002)	*TRAPPC9*	0.59 (<0.001)	0.56 (0.046)
*miR-93-5p*	0.92 (0.023)	1.03 (0.552)	*IGF2*	1.44 (<0.001)	not detected
*miR-106b-5p*	0.95 (0.042)	1.04 (0.830)	*–*	*–*	*–*
*miR-125a-5p*	1.17 (0.061)	0.99 (0.879)	*–*	*–*	*–*
*miR-125b-5p*	0.94 (0.072)	1.05 (0.599)	*ETS1*	1.48 (<0.001)	1.18 (0.509)
			*IGF2*	1.44 (<0.001)	not detected
			*TNFAIP3*	1.96 (<0.001)	2.14 (0.120)
*miR-181b-5p*	1.31 (<0.001)	1.12 (0.008)	*ASB13*	0.69 (<0.001)	0.64 (0.004)
			*SEC24C*	0.69 (<0.001)	0.62 (0.018)
*miR-425-5p*	1.27 (0.004)	1.28 (0.052)	*–*	*–*	*–*

*FC* Fold change

**Table 3:** Validation of deregulated miRNAs and correlating target mRNAs in HN2092 after radiochemotherapy treatment (analyzed with Agilent microarrays and TaqMan single qRT-PCR assays)

miRNA	Array	qRT-PCR	mRNA	Array	qRT-PCR
	FC (*p* value)	FC (*p* value)		FC (*p* value)	FC (*p* value)
*miR-93-5p*	0.96 (0.001)	0.84 (<0.001)	*CCDC88C*	1.44 (<0.001)	1.04 (0.890)
*miR-99b-5p*	1.13 (<0.001)	1.10 (0.502)	*ACTR1B*	0.62 (<0.001)	0.61 (0.012)
*miR-181a-5p*	1.38 (<0.001)	1.37 (0.001)	*–*	–	–
*miR-183-5p*	1.18 (<0.001)	1.18 (0.071)	*ASNS*	0.52 (<0.001)	0.47 (0.017)
			*IDH2*	0.63 (<0.001)	0.62 (0.045)
			*HSPA1B*	0.52 (0.022)	0.81 (0.167)
*miR-186-5p*	1.15 (0.083)	1.10 (0.147)	*FASN*	0.66 (<0.001)	0.66 (0.007)
*miR-197-3p*	1.17 (0.025)	1.02 (0.273)	*FASN*	0.66 (<0.001)	0.66 (0.007)
			*HSPA1B*	0.52 (0.022)	0.81 (0.167)
*miR-222-3p*	1.10 (0.006)	0.95 (0.057)	*FASN*	0.66 (<0.001)	0.66 (0.007)
*miR-320a*	1.14 (<0.001)	1.07 (0.425)	*HES6*	0.65 (<0.001)	0.52 (0.004)

*FC* Fold change

Furthermore, qRT-PCR analyses were performed in order to validate the network showing common interactions of HN1957 and HN2092 in response to radiochemotherapy-treatment (Fig. 5). Upregulation of *miR-7-5p* and *miR-17-5p* was verified for both cell lines as well as downregulation of their target genes *SNCA*, *AMD1*, *MKI67*, *BACE1* and NAGK in HN1957 or HN2092 (Table 4). Additionally, downregulation of *miR-93-5p* in HN2092 and upregulation of its target gene *SLC19A1* were verified (Table 4). Spearman correlation coefficients demonstrated a negative correlation (≤ −0.5) for five out of the eight miRNA-mRNA pairs of the combined network (Table 4)./

**Table 4:** Validation of miRNA-mRNA interactions in HN1957 and HN2092 in response to radiochemotherapy treatment (analyzed with TaqMan single qRT-PCR assays)

	miRNA	FC	mRNA	FC	Spearman correlation
HN1957	*miR-7-5p*	1.30	*SNCA*	0.48	0.5
	*miR-17-5p*	1.21	*AMD1*	0.87	−1.0
			*MKI67*	0.62	1.0
HN2092	*miR-7-5p*	1.54	*HELLS*	1.26	−0.5
	*miR-17-5p*	1.13	*BACE1*	0.96	−1.0
			*NAGK*	0.81	−0.5
	*miR-93-5p*	0.84	*CCDC88C*	1.04	0.5
			*SLC19A1*	2.38	−1.0

*FC* Fold change

### Characterization of the role of therapy-responsive circulating miRNAs on cellular pathways

As it was already shown by Summerer et al. [15], several miRNAs significantly deregulated in the presented radiochemotherapy cell culture model were also detectable as circulating deregulated miRNAs in HNSCC patients after radiochemotherapy. In order to gain information on the function of these therapy-responsive miRNAs (*miR-21-5p*, *miR-93-5p*, *miR-106b-5p* and *miR-425-5p*) all mRNAs that showed negatively correlating expression values (c ≤ −0.5) in the primary cell cultures and additionally representing validated targets in the miRTarBase were determined (Additional files 10 and 11). Pathway enrichment analysis (FDR < 0.05) of these potential target genes revealed predominantly signaling molecules that represent *direct P53 effectors* and play a role in *pathways in cancer*, *cell cycle* and the *E2F transcription factor network* (Tables 5 and 6). The key players of these pathways were *E2F1*, *PTEN*, *AKT2*, *JUN*, *HSP90AA1*, *KAT2B* in HN1957 and *JUN*, *KAT2B*, *BIRC5*, *CCND2, RBL2* in HN2092.

**Table 5:** Pathway enrichment analysis of potential target genes in HN1957 for miRNAs responding to therapy in HNSCC patients (FDR < 0.05)

Pathway	Number of Proteins in Pathway	Proteins from Gene List	*P*-value	FDR	Genes
Pathways in cancer (K)	327	7	0.0004	1.40E-02	E2F1,PTEN,AKT2,MSH6,HSP90AA1,JUN,VEGFA
Direct p53 effectors (N)	133	5	0.0002	1.40E-02	E2F1,PTEN,SP1,JUN,SMARCA4
Hepatitis B (K)	146	5	0.0004	1.38E-02	E2F1,PTEN,AKT2,JUN,YWHAQ
Nonsense-mediated decay (R)	106	5	0.0001	1.55E-02	RPL30,UPF1,SMG7,RNPS1,RPL18A
RNA transport (K)	164	5	0.0006	1.50E-02	EEF1A1,UPF1,RNPS1,EIF4G2,NUP205
E2F transcription factor network (N)	68	4	0.0002	1.44E-02	E2F1,KAT2B,SP1,RRM2
Estrogen signaling pathway (K)	100	4	0.0009	1.69E-02	AKT2,HSP90AA1,SP1,JUN
Glucocorticoid receptor regulatory network (N)	77	4	0.0003	1.34E-02	SMARCD1,HSP90AA1,JUN,SMARCA4
HIF-1-alpha transcription factor network (N)	66	4	0.0002	2.03E-02	NPM1,SP1,JUN,VEGFA
Huntington disease (P)	121	4	0.0017	2.79E-02	GAPDH,AKT2,AP2A2,JUN
Processing of capped intron-containing pre-mRNA (R)	138	4	0.0028	3.33E-02	DDX23,PCBP1,RNPS1,NUP205
Prostate cancer (K)	89	4	0.0006	1.56E-02	E2F1,PTEN,AKT2,HSP90AA1
Regulation of androgen receptor activity (N)	49	4	0.0001	1.70E-02	KAT7,HSP90AA1,KAT2B,JUN
Regulation of telomerase (N)	68	4	0.0002	1.44E-02	E2F1,HSP90AA1,SP1,JUN

(B) BioCarta, (K) KEGG Pathway, (N) NCI - Nature Curated Data, (P) pantherdb, (R) Reactome

**Table 6:** Pathway enrichment analysis of potential target genes in HN2092 for miRNAs responding to therapy in HNSCC patients (FDR < 0.05)

Pathway	Number of Proteins in Pathway	Proteins from Gene List	*P*-value	FDR	Genes
ISG15 antiviral mechanism(R)	71	4	0	<1.000E-03	NUP153,EIF4G2,NUP205,KPNA2
Viral carcinogenesis(K)	206	4	0.0006	1.94E-02	KAT2B,RBL2,CCND2,JUN
HTLV-I infection(K)	260	4	0.0013	3.58E-02	KAT2B,MAD2L1,CCND2,JUN
Aurora B signaling(N)	40	3	0.0001	7.00E-03	BIRC5,NPM1,PSMA3
Signaling events mediated by HDAC Class I(N)	56	3	0.0002	1.17E-02	NUP153,KAT2B,YY1
E2F transcription factor network(N)	68	3	0.0003	1.34E-02	KAT2B,RBL2,YY1
Validated targets of C-MYC transcriptional activation(N)	72	3	0.0003	1.35E-02	BIRC5,CCND2,NPM1
Mitotic Prophase(R)	99	3	0.0009	2.79E-02	NUP153,SET,NUP205
Nonsense-Mediated Decay(R)	106	3	0.0010	3.07E-02	SMG7,RPL30,RPL7
Cell cycle(K)	124	3	0.0016	3.55E-02	RBL2,MAD2L1,CCND2
Mitotic G1-G1/S phases(R)	134	3	0.0020	3.66E-02	RBL2,CCND2,PSMA3
Mitotic Metaphase and Anaphase(R)	173	3	0.0042	4.77E-02	BIRC5,MAD2L1,PSMA3

(B) BioCarta, (K) KEGG Pathway, (N) NCI - Nature Curated Data, (P) pantherdb, (R) Reactome

## Discussion

In the present study we applied an integrative approach for the delineation of the effects of radiochemotherapy on the molecular processes in a HNSCC cell culture model. Based on these data, we analyzed the cellular pathways affected by the treatment. The usefulness of this approach for the identification of regulatory networks has already been demonstrated in previous studies [19, 20]. For the first time we used this approach on a cell culture model with primary HNSCC cells mimicking a common therapy regime for HNSCC [4]. In this way we aimed for a better understanding of the treatment response, with respect to a common and individually varying molecular response.

The observed overlap of deregulated miRNAs and mRNAs between HN1957 and HN2092 hints to a partially common response to radiochemotherapy treatment. At the same time, the separation of the two primary HNSCC cell cultures in distinct clusters for both, the top 50 deregulated miRNAs (Fig. 1) and mRNAs (Fig. 2), suggests individual differences in the response to the treatment. This is consistent with differences between HN1957 and HN2092 in the sensitivity towards radiochemotherapy treatment as shown before [15]. The short-term effect on cellular viability following radiochemotherapy treatment became apparent only in HN1957, but not in HN2092 [15]. Variations in the treatment response may be attributed to inter-tumor heterogeneity among the various types of HNSCC [6] with regard to the tumor site and the molecular profile of the tumors [21]. Accordingly, array CGH detected alterations in both primary cultures that are typical for HNSCC, such as gains on 5p, 8q, 11q, 9q and 20pq as well as losses on 3p and 18q (reviewed in [22]) (Additional files 1, 2, 3A and 4A). In addition, alterations that were unique to one of the primary cell cultures, such as chromosomal bands on chromosomes 4 and 14, which were affected only in HN2092 but not in HN1957, were observed. Structural rearrangements involving chromosomes 1, 3, 8 and 13 that were detected in HN1957 or HN2092 are in accordance with previous karyotyping investigations of HNSCC [23]. In addition, so far unpublished rearrangements on chromosomes 4, 9, 10, 14 and 15 were discovered in the two primary cell cultures. Altogether, the cytogenetic analysis demonstrated that both primary cultures consisted of a rather homogenous cell population since most of the chromosomal alterations were shown to be clonal.

We further analyzed the mutational status of *P53* and *EGFR* since mutations in *P53* are common in head and neck cancers [24] and *EGFR* represents a key oncogene in HNSCC [25]. *EGFR* did not show any mutations in both primary cultures, whereas HN1957 showed a *P53* mutation in the SH3 ligand (P72R) and a nonsense mutation (Δ331) in the tetramerization domain (TD). The polymorphism at position 72 (P72R) in *P53* affects the interaction between some *P53* mutants and *P73*, a *P53* homologue that can transcriptionally activate *P53* target genes [26]. The binding ability of *P53* and *P73* affects the response to chemotherapy *in vitro*, which points to a possible impact of the polymorphism in codon 72 on the chemosensitivity of tumor cells. The second *P53* mutation (Δ331) leads to a truncated protein, due to a stop codon in the TD. Most of the mutations in the TD lead to defects in oligomerization of *P53*, DNA binding, stimulation of the transcription of reporter genes and growth inhibition of tumor cells [27]. It has been shown that deletion of the TD, which impairs the ability of *P53* to tetramerize, does not abolish its ability to bind DNA and to stimulate transcription, but significantly decreases the overall affinity of *P53* for DNA, thus destabilizing the *P53*-DNA complexes [28].

Since *EGFR* is known to be overexpressed in up to 90 % of HNSCC [29] we determined the *EGFR* expression levels of HN1957 and HN2092 in comparison to normal human keratinocytes (OKF6-hTERT). Both primary HNSCC cultures demonstrated increased relative expression of *EGFR* (Additional file 5), which implies a potential impact of *EGFR* signaling suggesting an *EGFR*-targeted treatment for an improved therapy response. Additionally, the epithelial cell adhesion molecule (*EpCAM*), which is frequently overexpressed in HNSCC [30], showed higher expression in HN1957 and HN2092 cells relative to OKF6-hTERT cells (Additional file 5). *EpCAM* acts as a marker for metastasis and proliferation representing another potential target for the therapeutic response.

The two primary cell cultures, HN1957 and HN2092, in part showed the same molecular response to radiochemotherapy treatment. The similarities became clear after pathway enrichment analysis that resulted in six common pathways affected by combined treatment with ionizing radiation and 5-FU. Among these, the pathway *direct effectors of P53* is likely to represent effects of both ionizing radiation and 5-FU treatment. 5-FU is known to stabilize and activate *P53* promoting *P53*-mediated apoptosis [31, 32]. *P53* is also activated by radiation-induced DNA-damage [33] and therefore represents an important cell cycle checkpoint. Another pathway affected by the treatment in our study was *apoptotic execution phase* including histones and molecules involved in DNA fragmentation and chromatin condensation. A further pathway, which was involved in the treatment response of both primary cell cultures, was *DNA damage/telomere stress induced senescence*. It is mainly based on histones that were damaged by the treatment and might reflect effects of ionizing radiation on the DNA structure. Molecules acting in the *cholesterol biosynthesis* also showed deregulation in both primary cell cultures, suggesting an involvement of membranes, probably due to an effect of 5-FU on lipids [34]. Further, the appearance of *dissolution of fibrin clot* as a result of the pathway analysis implies that cellular migration and inflammation was affected by the treatment since plasminogen activators and inhibitors regulate cellular adhesion and migration as well as inflammatory response [35]. *Unfolded protein response* was another pathway playing a role in the cellular treatment response due to deregulated chaperones, which are part of the cellular stress response [36]. Apart from that, we were able to validate deregulation of *miR-183-5p* and its target gene *ASNS* following radiochemotherapy (Table 3). Activation of *ASNS* transcription is part of the *unfolded protein response* and enhances the cellular resistance to drug treatment. Thus it represents a potential prognostic factor for the outcome of radiochemotherapy [37].

The miRNA-mRNA networks showing interactions that are part of the treatment response revealed three commonly deregulated miRNAs, *miR-7-5p*, *miR-17-5p* and *miR-93-5p*, in the two primary cell lines. For these miRNAs interactions with several target genes were validated for each primary cell line, which proves the significance of the treatment-responsive miRNA-mRNA interactions.

Genes, which are already known as key players in the response to 5-FU treatment, also appeared in the miRNA-mRNA networks of the two primary cell cultures. In particular, 5-FU is an anti-metabolite and inhibits thymidilate synthase, which catalyzes the synthesis of thymidylate and is an essential component of DNA replication and repair [31]. *SLC19A1* and *DHFR* are part of the folate metabolism, which is necessary for the reaction catalyzed by thymidilate synthase, and therefore they might represent predictive markers for the efficacy of 5-FU treatment [38]. Molecules, such as *ASNS* or *DHFR*, that show a response to treatment are potential candidates for stratification of patients with regard to their sensitivity to anti-tumor treatment and might be targets in a specific group of patients for a combinatorial treatment approach in order to enhance therapy success [7]. A systems-based prediction of such combinatorial treatment approaches has recently been reported for colon cancer by Klinger et al. [39], which would be also very promising in the case of HNSCC. Based on the current study such a systems analysis of HNSCC cells in response to additional inhibitors and perturbations becomes feasible.

Despite many common features, differences in the molecular treatment response between the two primary cultures were observed. The pathway enrichment analysis of treatment-responsive genes revealed pathways that were only affected in one of the two primary cell cultures. The *TGF-beta signaling pathway* was affected in HN1957 as well as the *regulation of nuclear SMAD2/3 signaling*. The two pathways are closely connected since both *SMAD2* and *SMAD3* are regulated by *TGF-beta* [40]. *TGF-beta* signaling is involved in cellular processes such as cell growth, cell differentiation and apoptosis. Further pathways that distinguished the response of the two primary cell cultures were *TNF signaling pathway* and *IL6-mediated signaling events*. These pathways are both part of inflammatory processes which might point to an immunological response to treatment in HN1957 cells. The cytokines *TGF-beta*, *IL6* and *TNF-alpha* are all well-known biomarkers for treatment complications and prognosis of radiochemotherapy success [41, 42]. Accordingly, the miRNA analysis revealed many miRNAs regulating immune response and inflammatory molecules deregulated in HN1957, but not in HN2092. For example, *miR-18a-5p*, *miR-106b-5p*, *miR-92a-3p* and *miR-125b-5p* are known to play a role in inflammation or immune system [43] and showed a treatment response only in HN1957. Moreover, upregulation of *miR-181b-5p* following treatment was validated in HN1957. *MiR-181b-5p* is an oncogenic miRNA known to be overexpressed in HNSCC and represents a previously reported link between inflammation and cancer [44]. Taking all these differences between HN1957 and HN2092 concerning the pathways involved in the molecular treatment response into account, some of the discovered pathways might be important for prognosis of the individual therapy success. As a consequence this novel knowledge may be used to deduce more individualized treatment strategies, e.g. targeting inflammatory pathways which might lead to a better treatment response [39].

The fact that four miRNAs (*miR-21-5p*, *miR-93-5p*, *miR-106b-5p*, *miR-425-5p*) that have already been shown to be therapy-responsive in blood plasma of HNSCC patients [15] were also deregulated in the cell culture model, demonstrates the clinical impact of this study and links the results of the cell culture model to our *in vivo* findings. Therefore, we identified all possible target molecules of these miRNAs by correlation analysis of miRNA and mRNA expression values, including only target interactions that are validated in the miRTarBase [17, 18]. The four miRNAs were previously described to play a role in cancer and represent potential diagnostic or prognostic biomarkers. *MiR-106b-5p* and *miR-21-5p* were suggested as biomarkers in laryngeal carcinoma [45]. Moreover, *miR-106b-5p* has been shown to promote cell migration and invasion by targeting *PTEN* [46] while *miR-21-5p* is overexpressed in various cancer types and was reported as a prognostic biomarker in head and neck cancer [47]. *MiR-425-5p* and *miR-93-5p* are known as regulators in cell proliferation [48, 49]. *MiR-93-5p* is also targeting the *PTEN/AKT* signaling pathway, thus influencing drug sensitivity of cancer cells [50]. The pathway enrichment analysis based on the target genes of these miRNAs revealed mostly signaling molecules that represent *direct P53 effectors* such as *PTEN*, *JUN* and *E2F1* as well as *cell cycle* regulators such as *RBL2*, *CCND2*, *RRM2* and *E2F1*. The *E2F transcription factor network* including genes such as *E2F1*, *RRM2*, *RBL2*, *KAT2B* represents a crucial target of the four selected miRNAs in both primary HNSCC cultures, which might be due to the fact that *E2F1* impacts thymidilate synthase expression, which is a major target of 5-FU as already discussed [51]. Furthermore, several studies report an influence of deregulation of the *E2F transcription factor network* on the chemoradiation sensititvity of cancer cells [52–54]. Most of the genes, that are involved in many of the significantly enriched pathways, also play a role in *pathways in cancer* such as *PTEN*, *JUN*, *AKT2*, *HSP90AA1*, the latter of which was already described to influence radiosensitivity and chemosensitivity [55]. Also *PTEN* is a well-known radiosensitizer enhancing cell death through *AKT* signaling [56]. The results presented in this study open up the possibility of new treatment strategies that target the therapy-responsive signaling pathways either directly or on the level of the miRNAs regulating the signaling molecules.

## Conclusions

Important progress in strategies for treatment of HNSCC has been made over the past decades, however, dose escalation studies revealed that classical radiochemotherapy has reached some sort of dead end [57]. Therefore, a combination of radiochemotherapy with molecularly targeted agents might open up new therapeutic possibilities. This requires the identification of prognostic targets that enable individualized treatment strategies and allow prevention of excessive therapy.

In the present study we showed that the main pathways affected by radiochemotherapy in two different HNSCC primary cultures are related to cell cycle and proliferation, cell death and stress response. As a difference between the two cell cultures we discovered an emphasis on inflammation in the treatment response of HN1957. This suggests the use of inflammatory pathways for stratification of HNSCC patients in order to identify individuals who might benefit from an additional therapy targeting inflammatory pathways.

Similar pathways emerged from the analysis of potential targets of four miRNAs that showed a treatment response in the plasma of HNSCC patients and the cell culture model, suggesting potential molecular therapeutic targets in the *E2F transcription factor network* and the *PTEN/AKT* signaling pathway. This leads to the conclusion that promising prognostic markers and molecules for a targeted therapy approach in HNSCC patients are most likely to be found among those signaling molecules which needs to be further investigated on clinical samples.

## Methods

### Primary HNSCC cell cultures

The primary HNSCC cell cultures, HN1957 and HN2092, were previously described by Summerer et al. [15]. Characteristics of the two primary cell cultures are listed in Table 1. Molecular characterization of the primary cell cultures included array CGH, SKY, sequence analysis of *TP53* and *EGFR* and determination of EGFR and EpCAM protein expression levels on the cell surface.

#### High-Resolution Oligo Array CGH

For array CGH analysis of the primary cell cultures the SurePrint G3 human CGH Microarray Kit 4x180k (Agilent Technologies, Santa Clara, CA, AMADID: 022060) was used. Tumor DNA (250 ng) and sex-mismatched normal reference DNA (250 ng) (Promega, Madison, WI) were used for hybridization. Hybridization and data analysis were performed as described by Hess et al. [58].

#### SKY

Metaphase preparation was done with 3 h of colcemid (Roche) treatment followed by hypotonic treatment with KCl (75 mM) for 25 min and three fixation steps (20 min each) with methanol-acetic acid (3 + 1) on ice. After one week of ageing at room temperature metaphase preparations were treated with RNase A (50 μg/mL in 2 x SSC), digested with pepsin (1 mg/ml) for 2 min at 37 °C and dehydrated in a 70, 80, and 100 % ethanol series. After fixation with 1 % formaldehyde for 10 min metaphases were placed in denaturing solution (70 % formamide in 2 x SSC) at 72 °C for 7 min followed by dehydration. Hybridization steps and image analysis were previously described by Hieber et al. [59].

#### Sequencing

Complementary DNA was synthesized from cellular RNA using the SuperScript III First-Strand Synthesis System for RT-PCR (Invitrogen, Carlsbad, CA) according to the manufacturer’s protocol using Oligo-dT primer and 1.6 μg RNA. Subsequently, PCR was performed using Q5 High-Fidelity DNA Polymerase (New England Biolabs, Ipswich, MA) with 1 μl of a 1:10 dilution of the cDNA using the primer combinations in Additional file 12. The protocol was optimized for a 50 μl reaction volume using 1 μl forward and 1 μl reverse primer and adding 5 μl of 10× cresol red and 1 μl of DMSO. The PCR was optimized as follows: denaturation for 10 min at 96 °C followed by 35 cycles of 15 s at 96 °C and 8 min at 68 °C and final extension for 10 min at 68 °C. The size of the PCR-products was checked on a 1 % agarose gel. The bands were cut out from the gel and DNA was purified on spin columns. The following BigDye PCR was performed using the BigDye Terminator V3.1 Kit (Applied Biosystems, Waltham, MA) with 6 μl of template DNA. PCR was carried out with 4 min of denaturation at 96 °C followed by 45 cycles of 30 s 95 °C, 20 s 50 °C and 4 min 60 °C. PCR products were sequenced on an ABI 3730 DNA Analyzer (Applied Biosystems, Waltham, MA).

#### EGFR and EpCAM expression

Surface expression levels of EGFR and EpCAM were assessed by flow cytometry using fluorescently labeled antibodies as described before [60]. Briefly, 1×10^5^ cells were stained with anti-EGFR-PE (clone EGFR.1) and anti-EpCAM-APC (clone EBA-1) antibodies or the corresponding isotype controls (all from BD Biosciences, Franklin Lakes, NJ) in PBS supplemented with 2 % FCS for 20 min at 4 °C. Cells were washed twice and analyzed on an LSRII flow cytometer (BD Biosciences, Franklin Lakes, NJ). Relative surface expression levels are depicted as median fluorescence intensities subtracted by the matching isotype controls (means ± standard deviations of 3 technical replicates are given). Expression of EGFR and EpCAM was measured for HN1957 and HN2092 as well as for the immortalized keratinocytes OKF6-hTERT [61].

### Treatment of HNSCC cells

The treatment of the primary HNSCC cells was designed to model radiochemotherapy treatment of a HNSCC patient cohort used in a previous study by Summerer et al. [15]. Briefly, cells were irradiated with 2 Gy using a ^137^Cs source and treated with 5-FU (solved in DMSO; Sigma-Aldrich, St. Louis, MO). Controls were treated with the corresponding volumes of DMSO and sham-irradiated. 24 h after the first irradiation a second fraction of 2 Gy was applied to the 5-FU-treated cells followed by incubation for 1 h at 37 °C. Cells were harvested by trypsinization and stored at −20 °C until further processing.

### RNA extraction and quality assessment

Total RNA was extracted from frozen cell pellets (−20 °C) of treated and untreated primary HNSCC cells using the miRNeasy mini kit (Qiagen, Venlo, Netherlands) according to the manufacturer’s protocol without DNase digest or small RNA enrichment. Optical density (OD) 260/280 ratios were measured with a Nanodrop ND-1000 (Thermo Scientific) and ranged from 1.92 to 2.04. RNA-concentrations were measured with a Qubit 2.0 Fluorometer (Invitrogen, Carlsbad, CA) using the RNA Broad Range Assay Kit (Invitrogen, Carlsbad, CA). Additionally, RNA quality was assessed prior to the Agilent microarray experiments using an Agilent 2100 Bioanalyzer (Agilent Technologies, Santa Clara, CA). The obtained RNA integrity numbers (RINs) ranged from 9.3 to 10.0. RNA samples were stored at −80 °C until further processing.

### MicroRNA profiling

MiRNA profiling of primary HNSCC cell cultures was previously described by Summerer et al. [15].

### Quantification of individual miRNAs by real-time PCR

Reverse transcription was performed on a Cyclone PCR system (Peqlab, Erlangen, Germany) using the TaqMan miRNA reverse transcription kit and miRNA-specific stem-loop primers (Applied Biosystems, Waltham, MA) according to the manufacturer’s protocol. Quantitative real-time PCR (qRT-PCR) was performed in duplicates and included non-template negative controls. A ViiA 7 real-time PCR System (Applied Biosystems, Waltham, MA) was used according to the manufacturer’s protocol. The U6 snRNA was used for normalization. Fold changes were calculated using the 2^-ΔΔCt^ method [62]. *P*-values were computed using the student’s *t*-test.

### Global gene expression analysis

To identify potential targets of deregulated miRNAs, a gene expression profiling was performed with G3 Human Gene Expression 8×60k v2 microarrays (Agilent Technologies, Santa Clara, CA, AMADID: 039494) covering over 40,000 transcripts. The gene expression analysis was carried out according to the manufacturer’s protocol. Total RNA was extracted from untreated and treated cells as described above. A one-color microarray experiment with 60 ng of the same RNA samples that were used for the miRNA analysis was conducted with three biological and two technical replicates for each data point. A one-color RNA spike-in kit (Agilent Technologies, Santa Clara, CA) was used to monitor the workflow. In the first step copyDNA (cDNA) was generated from the RNA templates followed by transcription to copyRNA (cRNA) with incorporation of cyanine 3-CTP. After hybridization of the labeled cRNA on the arrays (17 h, 65 °C), the microarrays were scanned with a G2505C Sure Scan Microarray Scanner (Agilent Technologies, Santa Clara, CA). Data were extracted with the Feature Extraction 10.7 software (Agilent Technologies, Santa Clara, CA). Data quality assessment, preprocessing, and normalization were conducted in R using the Bioconductor AgiMicroRNA package [63]. In order to identify significantly differentially expressed genes between treated and untreated cells, statistical analyses were accomplished using the Bioconductor limma package [64]. A cut-off for FDR-adjusted *p*-values of 0.05 was applied.

### Quantitative real-time PCR quantification of individual mRNAs

For validation of gene expression microarray data, individual mRNAs were quantified via qRT-PCR. 500 ng of RNA was reverse-transcribed using the QuantiTect Reverse Transcription Kit (Qiagen, Venlo, Netherlands) according to the manufacturer’s protocol. qRT- PCR was performed on a ViiA 7 real-time PCR System (Applied Biosystems, Waltham, MA) using specific TaqMan gene expression assays (Applied Biosystems, Waltham, MA). PCR was carried out in 10 μl reactions consisting of 5 μl TaqMan PCR Master Mix (no AmpErase UNG), 3.5 μl H_2_O, 0.5 μl TaqMan assay and 1 μl cDNA. All reactions were performed in triplicates and included non-template negative controls. *B2M* and *ACTB* were used as endogenous controls. Fold changes were calculated using the 2^-ΔΔCt^ method [62]. *P*-values were computed using the student’s *t*-test.

### Network analysis

MiRNA-mRNA networks were designed based on integrative analysis of the microarray data. A correlation matrix was calculated using the expression values of all significantly deregulated miRNAs and mRNAs, resulting in a correlation value for each miRNA-mRNA pair. Based on the assumption of a negative regulation mechanism Pearson correlation values of −1 ≤ c ≤ −0.5 were considered to indicate associations. This condition was used to convert the correlation matrix into a binary matrix to which we associated a false detection rate calculated after a permutation test. Only miRNA-mRNA pairs that represented validated interactions (with the annotation “strong evidence” or NGS-validated targets) in the miRTarBase [18, 17], were considered. The miRNA-mRNA pairs that showed significant negative correlation as well as validated functional interaction (according to miRTarBase) were visualized with the yED Graph Editor software [65]. All statistical analyses were performed using the R Project for Statistical Computing [66].

### Pathway analysis of deregulated mRNAs

To analyze the functional context of the significantly deregulated genes in the radiochemotherapy cell culture model a pathway enrichment analysis was performed using the Reactome 4.0.1 application [14] in the Cytoscape 3.0.2 software [67].

## Additional files

Additional file 1:
**Copy number alterations in HN1957.** (PDF 37 kb) Additional file 2:
**Copy number alterations in HN2092.** (PDF 43 kb) Additional file 3:
**Cytogenetic characterization of HN1957.** (PDF 8257 kb) Additional file 4:
**Cytogenetic characterization of HN2092.** (PDF 8434 kb) Additional file 5:
**EGFR and EpCAM surface expression of HN1957, HN2092 and OKF6-hTERT.** (PDF 838 kb) Additional file 6:
**Significantly deregulated mRNAs in primary HN1957 after** ***in vitro*** **radiochemotherapy treatment.** (PDF 146 kb) Additional file 7:
**Significantly deregulated mRNAs in primary HN2092 after** ***in vitro*** **radiochemotherapy treatment.** (PDF 143 kb) Additional file 8:
**Pathway enrichment analysis of differentially expressed genes in HN1957 after radiochemotherapy treatment.** (PDF 66 kb) Additional file 9:
**Pathway enrichment analysis of differentially expressed genes in HN2092 after radiochemotherapy treatment.** (PDF 44 kb) Additional file 10:
**Potential target genes in HN1957 for miRNAs responding to therapy in HNSCC patients.** (PDF 72 kb) Additional file 11:
**Potential target genes in HN2092 for miRNAs responding to therapy in HNSCC patients.** (PDF 51 kb) Additional file 12:
**Primer sequences for** ***TP53*** **and** ***EGFR*** **sequencing.** (PDF 42 kb)

## Abbreviations

^137^Cs: ^137^Cesium
5-FU: 5-fluorouracil
APC: Allophycocyanin
CGH: Comparative genomic hybridization
cRNA: copyRNA
CTP: Cytosine triphosphate
DMSO: Dimethyl sulfoxide
EBA-1: EpCAM antibody 1
EBV: Ebstein Barr virus
FCS: Fetal calf serum
FDR: False discovery rate
HNSCC: Head and neck squamous cell carcinoma
HPV: Human papilloma virus
hTERT: Human telomerase reverse transcriptase
KCl: Potassium chloride
miRNA: microRNA
mRNA: messengerRNA
NGS: Next generation sequencing
OD: Optical density
PBS: Phosphate buffered saline
PCR: Polymerase chain reaction
PE: Phycoerythrin
qRT-PCR: Quantitative real-time PCR
SKY: Spectral karyotyping
snRNA: small nucleolar RNA
SSC: Saline sodium citrate
TD: Tetramerization domain
UNG: Uracil N-glycosylase
