# Supplementary material for: Integrative analysis of the microRNA-mRNA response to radiochemotherapy in primary head and neck squamous cell carcinoma cells
Source: BMC Genomics. 2015 Sep 2;16(1):654. doi: 10.1186/s12864-015-1865-x (PMC4557600; doi:10.1186/s12864-015-1865-x)
Supplement: Additional file 1: — Copy number alterations in HN1957. (PDF 37 kb) [file 12864_2015_1865_MOESM1_ESM.pdf]

**Additional file 1 Copy number alterations in HN1957**

| Chromosome | Location | Start (bp) | End (bp) | Size (Mb) | Gain/loss |
|---|---|---:|---:|---:|---|
| 1 | p31.3 | 64137196 | 64574378 | 437 | Loss |
| 1 | q21.1 | 145009491 | 145291681 | 282 | Loss |
| 1 | q21.2 | 147404182 | 149243878 | 1840 | Loss |
| 2 | p22.3 | 34702900 | 34714858 | 12 | Loss |
| 2 | p11.2 - 11.1 | 89427335 | 91815649 | 2388 | Loss |
| 2 | q21.2 | 133817872 | 134013314 | 195 | Loss |
| 3 | p26.3 - 11.1 | 73884 | 90282026 | 90208 | Loss |
| 5 | p15.33 - 12 | 26112 | 45872739 | 45847 | Gain |
| 5 | q35.3 | 177059894 | 178434885 | 1375 | Loss |
| 7 | p22.3 - 11.2 | 830127 | 56021762 | 55192 | Gain |
| 8 | p23.3 - 23.1 | 161442 | 7786619 | 7625 | Loss |
| 8 | p11.22 | 39237408 | 39345390 | 108 | Loss |
| 8 | q11.1 - 24.3 | 46943427 | 146294012 | 99351 | Gain |
| 9 | p24.3 - q21.13 | 204163 | 75759274 | 75555 | Gain |
| 9 | q21.13 | 75773390 | 75785187 | 12 | Gain |
| 9 | q21.13 - 34.3 | 75793096 | 139521304 | 63728 | Gain |
| 10 | p15.3 - 11.21 | 136331 | 37912674 | 37776 | Loss |
| 11 | p15.5 - q25 | 210270 | 134927025 | 134717 | Gain |
| 12 | p11.22 - q13.11 | 29394531 | 47149663 | 17755 | Gain |
| 13 | q21.1 - 34 | 56908708 | 115105208 | 58197 | Gain |
| 15 | q11.1 - 11.2 | 20102511 | 22409302 | 2307 | Loss |
| 15 | q11.2 - 26.3 | 22425868 | 102480799 | 80055 | Gain |
| 16 | p13.3 - q24.3 | 106241 | 90163040 | 90057 | Gain |
| 17 | q11.1 - 25.3 | 25403416 | 81098955 | 55696 | Gain |
| 18 | p11.32 - q23 | 118730 | 78009943 | 77891 | Loss |
| 20 | p13 - q13.33 | 67748 | 62949060 | 62881 | Gain |
| 21 | p11.2 - q11.2 | 9832418 | 15499817 | 5667 | Loss |
| 21 | q11.2 - 21.1 | 15513138 | 17895158 | 2382 | Gain |
| 22 | q11.21 - 12.1 | 17927733 | 28915008 | 10987 | Gain |
| 22 | q12.2 - 13.33 | 31001795 | 51218920 | 20217 | Gain |
