# Supplementary material for: Integrative analysis of the microRNA-mRNA response to radiochemotherapy in primary head and neck squamous cell carcinoma cells
Source: BMC Genomics. 2015 Sep 2;16(1):654. doi: 10.1186/s12864-015-1865-x (PMC4557600; doi:10.1186/s12864-015-1865-x)
Supplement: Additional file 2: — Copy number alterations in HN2092. (PDF 43 kb) [file 12864_2015_1865_MOESM2_ESM.pdf]

Additional file 2 Copy number alterations in HN2092

Chromosome	Location	Start (bp)	End (bp)	Size (Mb)	Gain/loss
1	p34.1	45638843	46411830	773	Loss
2	p11.2 - 11.1	89129502	91815649	2686	Loss
3	p26.3 - 14.2	73884	60431612	60358	Loss
3	p14.2	60445716	60856997	411	Loss
3	p14.2	60877532	61256461	379	Loss
3	p14.2 - 14.1	61279362	65309302	4030	Gain
3	p14.1 - 13	65329274	71064421	5735	Gain
3	p12.1	85375309	85527808	152	Loss
4	p16.3 - 11	45852	49032819	48987	Loss
4	q13.2	69392515	69438151	46	Gain
4	q32.1	159836338	160477505	641	Gain
5	p15.33 - 12	26112	45872739	45847	Gain
5	q15	92922745	92925263	3	Loss
7	q21.11 - 31.1	85927890	111030944	25103	Gain
7	q31.1	111044005	111303942	260	Gain
7	q31.1 - 31.31	111316562	120348264	9032	Gain
7	q31.31 - 36.3	120355583	159118477	38763	Loss
8	p23.3 - 23.2	161442	3379024	3218	Loss
8	p23.2	3395242	3614215	219	Loss
8	p23.2	3623018	4532958	910	Loss
8	p12 - 11.23	30428574	37449311	7021	Gain
8	p11.23 - 11.22	37498547	39222337	1724	Gain
8	p11.22	39237408	39345390	108	Loss
8	p11.22	39392445	39587449	195	Gain
8	p11.22 - q24.3	39607186	146294012	106687	Gain
9	p24.3 - q13	204163	68452996	68249	Loss
9	q21.11 - 34.3	70984451	141018895	70034	Gain
11	q13.2 - 25	68090440	134927025	66837	Gain
12	q21.31 - 24.33	83072415	133291385	50219	Gain
13	q11 - 14.3	19296514	51173255	31877	Loss
13	q14.3 - 31.2	51212860	89898466	38686	Gain
14	q11.2	19376732	20414143	1037	Loss
14	q11.2	20465917	22428569	1963	Loss
14	q11.2 - 12	23016509	25424295	2408	Loss
14	q12 - 24.3	25443991	75707870	50264	Gain
14	q24.3	75727607	78086326	2359	Gain
14	q24.3 - 32.33	78104995	107278681	29174	Gain
16	p13.3	6754631	6982630	228	Loss

16	p11.2 - q24.3	29652969	90163040	60510	Gain
17	q25.3	80111187	81098955	988	Loss
18	p11.32 - q23	118730	78009943	77891	Loss
19	q13.2 - 13.43	42737047	59092485	16355	Gain
20	p13 - q13.33	67748	62949060	62881	Gain
21	p11.2	9832418	15347165	5515	Loss
22	q11.23	24347929	24390165	42	Loss
22	q11.23 - 12.1	25664588	25911562	247	Loss
