# Supplementary material for: Integrative analysis of the microRNA-mRNA response to radiochemotherapy in primary head and neck squamous cell carcinoma cells
Source: BMC Genomics. 2015 Sep 2;16(1):654. doi: 10.1186/s12864-015-1865-x (PMC4557600; doi:10.1186/s12864-015-1865-x)
Supplement: Additional file 4: — Cytogenetic characterization of HN2092. (PDF 8434 kb) [file 12864_2015_1865_MOESM4_ESM.pdf]

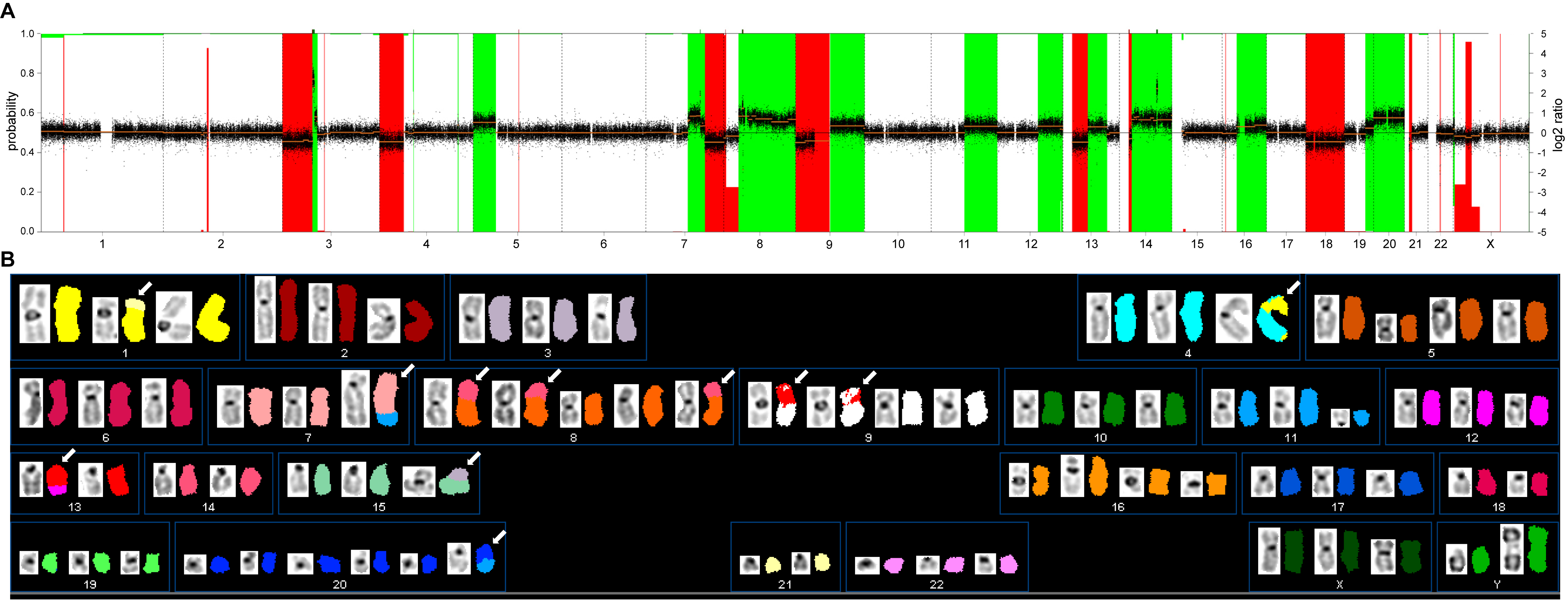

Cytogenetic characterization of HN2092. (A) The array CGH profile shows copy number alterations on several chromosomes. Green bars (top down) represent copy number gains at the corresponding position in the genome. Red bars (bottom up) indicate copy number losses. Bars reaching beyond the middle axis (probability >0.5) were called as gains or losses. (B) The spectral karyotype of a representative metaphase reveals various chromosomal alterations: 75,XY,+X,+del(X)(p21→pter),+i(X)(q10),+i(Y)(q10),+der(1)t(1;21)(p11→qter;qter→q11),+2,+3,+der(4)t(1;4)(pter→q21;?),+5,+i(5)(p10),+6,+der(7)add(7)(q31)t(7;11),+3xder(8)t(8;14)(p11→qter;qter→q11),+2xder(9)t(9;13)(p11→qter;qter→q14),+10,+del(11)(q11),+12,der(13)t(12;13)(?;p13→q22),+der(15)t(3;15)(?;p11→qter),+16,+der(16)(?),+17,+19,+3x20,+der(20)t(11;20)(?),+22. White arrows indicate chromosomes with color junctions.
