# Supplementary figures and images for: Integrative analysis of the microRNA-mRNA response to radiochemotherapy in primary head and neck squamous cell carcinoma cells
Source: BMC Genomics. 2015 Sep 2;16(1):654. doi: 10.1186/s12864-015-1865-x (PMC4557600; doi:10.1186/s12864-015-1865-x)

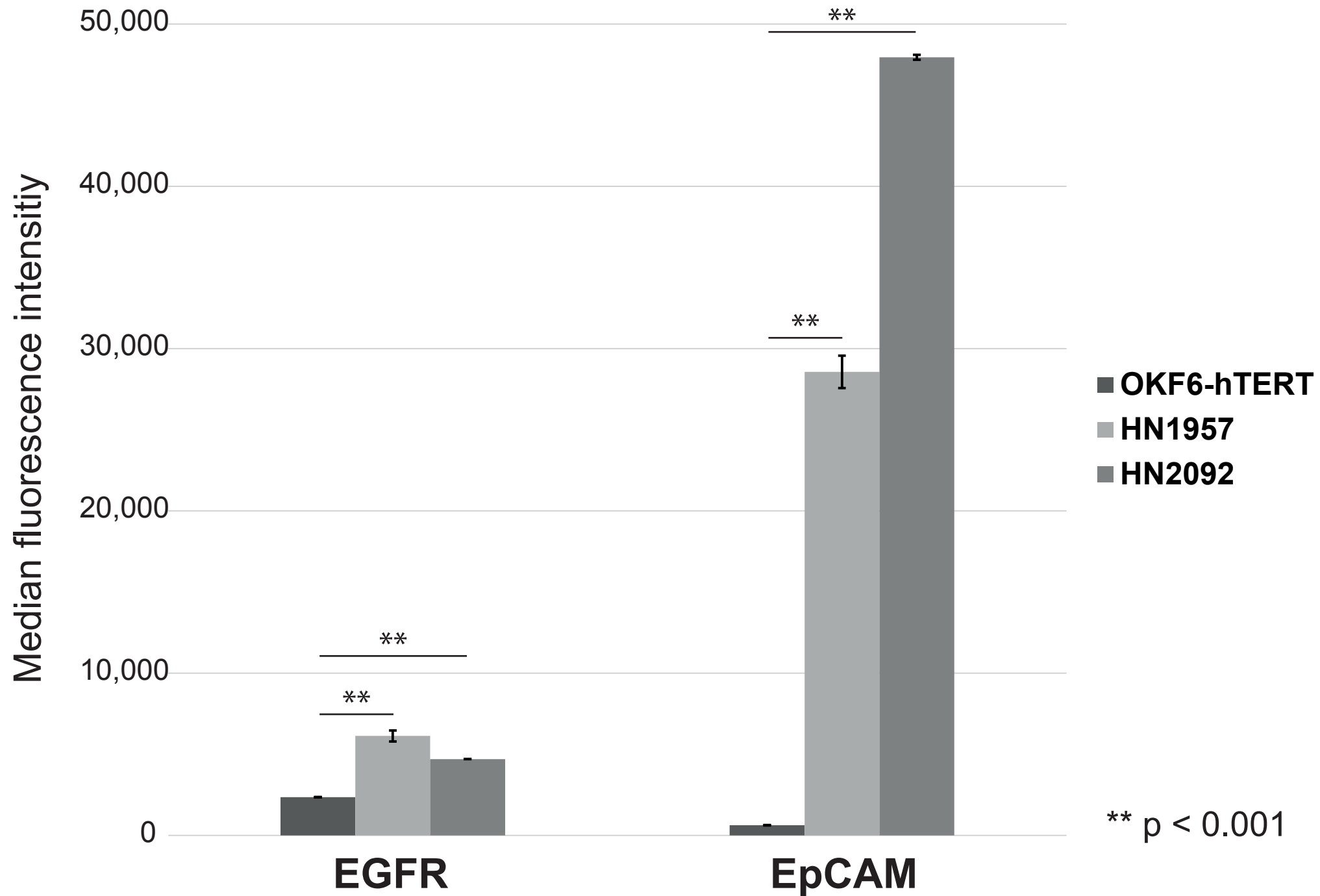

Supplement: Additional file 5: — EGFR and EpCAM surface expression of HN1957, HN2092 and OKF6-hTERT. (PDF 838 kb) [file 12864_2015_1865_MOESM5_ESM.pdf]
