# Supplementary material for: Integrative analysis of the microRNA-mRNA response to radiochemotherapy in primary head and neck squamous cell carcinoma cells
Source: BMC Genomics. 2015 Sep 2;16(1):654. doi: 10.1186/s12864-015-1865-x (PMC4557600; doi:10.1186/s12864-015-1865-x)
Supplement: Additional file 6: — Significantly deregulated mRNAs in primary HN1957 after in vitro radiochemotherapy treatment. (PDF 146 kb) [file 12864_2015_1865_MOESM6_ESM.pdf]

Additional file 6 Significantly deregulated mRNAs in HN1957 after radiochemotherapy treatment (adjusted p-value<0.05)

Gene	adjusted p-value	fold-change
TXNIP	6.35E-13	0.31
C8orf55	7.54E-12	2.19
RHBDF2	7.54E-12	1.96
IFIT1	7.54E-12	0.49
SNRPA1	7.54E-12	0.45
IFIT2	7.54E-12	0.31
SDF2L1	1.17E-11	1.91
OASL	1.17E-11	0.33
IL24	1.17E-11	0.28
MANF	1.27E-11	2.21
CTSC	1.84E-11	0.51
SLFN11	2.21E-11	2.13
IFRD1	2.23E-11	0.52
LHPP	2.62E-11	2.04
ARRDC4	2.62E-11	0.45
AGR2	2.81E-11	3.01
E2F2	3.08E-11	1.82
HMGA2	3.08E-11	0.46
GADD45A	3.94E-11	0.53
HSPA1A	4.72E-11	2.20
CALR	4.72E-11	1.81
RPL28	4.72E-11	0.45
TNFSF9	4.96E-11	2.25
KREMEN1	4.96E-11	1.79
INF2	7.46E-11	1.91
FADS2	8.67E-11	2.32
TET1	9.49E-11	2.36
C6orf48	9.49E-11	0.52
IFIT3	1.30E-10	0.50
HSPA1B	1.39E-10	1.96
DDX58	1.39E-10	0.52
BIRC3	1.39E-10	0.45
MVD	1.57E-10	2.31
FAM20C	1.65E-10	1.84
INSIG1	1.67E-10	2.32
LSS	1.67E-10	2.25
TMEM97	1.67E-10	2.01

OSBPL5	1.67E-10	1.95
CDK2AP2	1.67E-10	1.66
ANTXR2	1.67E-10	0.53
CH25H	1.67E-10	0.26
ABCB6	2.03E-10	1.66
ADAMTS6	2.03E-10	0.55
HIST1H2AG	2.65E-10	2.08
MEGF8	2.73E-10	1.89
LAMA3	2.77E-10	0.57
MAST1	2.95E-10	1.72
C6orf1	2.95E-10	1.58
IL23A	3.91E-10	0.58
HS3ST1	4.00E-10	0.60
ISG15	4.15E-10	0.64
TMEM79	4.15E-10	0.57
DDIT3	4.15E-10	0.52
B3GAT3	5.30E-10	1.55
COMTD1	5.34E-10	1.90
RGS2	5.34E-10	0.54
RUNX1	5.34E-10	0.45
PRR7	5.81E-10	1.63
HSP90B1	5.87E-10	1.67
CIDEC	6.29E-10	0.62
CRELD2	6.62E-10	1.69
PIF1	6.69E-10	1.71
TMEM187	6.91E-10	2.08
ETS2	7.04E-10	0.55
CMTM4	7.05E-10	1.61
ISG20	7.05E-10	0.64
SERPINB2	7.05E-10	0.39
PRMT7	7.26E-10	1.63
HR	7.26E-10	0.52
CBX6	7.35E-10	1.73
DUSP12	7.47E-10	0.63
ST6GALNAC2	7.97E-10	2.39
MYLK	8.49E-10	1.53
SEC24D	8.58E-10	1.82
CKLF	8.95E-10	1.61
FYB	8.95E-10	0.57
NLRP3	9.00E-10	0.53
LRP8	9.06E-10	1.93

PSKH1	1.04E-09	1.75
MYO5C	1.04E-09	1.73
NUAK2	1.04E-09	0.60
JDP2	1.10E-09	1.97
PLAU	1.10E-09	0.60
RPL10A	1.13E-09	1.47
HSD11B1	1.34E-09	0.48
XBP1	1.37E-09	1.68
BRD4	1.37E-09	0.66
NOG	1.37E-09	0.55
CELSR1	1.40E-09	1.55
FAM83A	1.40E-09	0.61
ANGPTL4	1.63E-09	0.61
DUX4	1.67E-09	1.60
PLEKHA6	1.81E-09	1.61
HTRA1	1.81E-09	1.58
PDLIM5	1.81E-09	0.69
BMP2	1.81E-09	0.65
UBAP1	1.81E-09	0.65
GADD45B	1.87E-09	0.57
PSAT1	2.08E-09	1.65
LAMB3	2.08E-09	0.60
SNN	2.16E-09	1.52
SP5	2.26E-09	1.62
C15orf42	2.26E-09	1.51
NKX2-5	2.29E-09	1.55
SGK1	2.29E-09	0.62
ZC3HAV1	2.31E-09	0.61
TST	2.39E-09	1.71
EDN1	2.42E-09	0.64
APOL6	2.66E-09	1.49
PCSK9	2.70E-09	1.80
HSD17B8	2.76E-09	1.55
BAIAP2	2.76E-09	0.69
SFI1	2.95E-09	1.64
C22orf40	3.22E-09	1.55
PSMG4	3.22E-09	1.50
IGFBP3	3.22E-09	0.59
NT5C3	3.24E-09	0.68
LFNG	3.29E-09	2.08
ZBED2	3.94E-09	0.61

RBM14	3.94E-09	0.63
B4GALNT1	4.01E-09	1.84
PHACTR3	4.04E-09	0.59
PHF13	4.10E-09	1.72
IMPA2	4.10E-09	1.61
TRAPPC9	4.45E-09	1.70
IDE	4.45E-09	1.54
PTGS2	4.45E-09	0.52
FGFR3	4.47E-09	1.66
METTL3	4.78E-09	1.53
ALDH4A1	5.06E-09	1.64
SYTL1	5.11E-09	1.57
TRIOBP	5.33E-09	1.82
MMD	5.33E-09	1.45
PNPLA3	5.42E-09	1.64
RNF145	5.49E-09	1.59
GTPBP5	5.49E-09	0.66
RTN4R	5.52E-09	1.56
EVL	5.66E-09	1.46
SPTLC2	5.84E-09	1.72
PRDM1	5.84E-09	0.51
FXYD3	6.19E-09	0.60
IDH1	6.45E-09	1.45
ASF1B	6.71E-09	1.64
PLAUR	6.71E-09	0.56
FGD3	6.83E-09	1.69
CYP27B1	6.83E-09	0.50
TUFT1	6.94E-09	0.70
RNF43	6.94E-09	0.63
TMEM40	6.96E-09	0.59
ANKRD52	7.00E-09	1.54
HES2	7.00E-09	0.64
C12orf23	7.11E-09	1.47
PTPN18	7.28E-09	1.43
HSPA8	7.77E-09	1.56
SLC6A9	7.80E-09	1.56
MYEOV	7.80E-09	0.63
CALB1	7.80E-09	0.60
LEPREL2	7.98E-09	1.55
HYAL2	7.98E-09	1.47
CYP27C1	8.23E-09	0.63

ATG16L2	8.46E-09	1.58
SERPINE1	8.57E-09	0.52
SLC48A1	8.79E-09	1.42
VEGFC	8.85E-09	0.69
IBA57	9.07E-09	0.55
CBX2	1.02E-08	1.54
PGLS	1.03E-08	1.49
ELFN2	1.03E-08	1.48
HSPG2	1.08E-08	1.64
PMAIP1	1.09E-08	0.60
SPC25	1.11E-08	1.54
MOB3B	1.11E-08	0.67
B9D1	1.12E-08	1.42
CAMKK1	1.14E-08	1.84
DUX4L4	1.14E-08	1.72
APOBEC3F	1.17E-08	1.58
NLRP2	1.18E-08	1.46
INHBA	1.18E-08	0.55
ANP32E	1.22E-08	1.56
TRIM8	1.25E-08	0.66
PANK1	1.30E-08	1.76
ZNF502	1.41E-08	0.64
HMGCR	1.42E-08	1.57
EVI5L	1.44E-08	1.80
SLC17A5	1.44E-08	1.54
LMNB1	1.44E-08	1.51
HES6	1.48E-08	1.57
HSBP1L1	1.50E-08	0.66
TMBIM4	1.51E-08	1.42
ADAMTS1	1.51E-08	0.61
IL1A	1.55E-08	0.66
NUDT8	1.60E-08	1.55
ARHGAP27	1.60E-08	0.69
MVK	1.68E-08	1.57
ITFG3	1.68E-08	1.43
MICA	1.72E-08	1.46
SMARCC2	1.72E-08	1.42
FADS1	1.72E-08	1.60
PCYOX1L	1.83E-08	1.46
HIST1H4L	1.88E-08	1.45
DHCR7	1.93E-08	1.49

SMOX	1.93E-08	0.63
MRPS30	1.98E-08	0.68
VNN1	2.00E-08	0.64
MCM6	2.05E-08	1.45
FLRT3	2.13E-08	0.62
XXYLT1	2.17E-08	1.45
FAT1	2.17E-08	0.70
ACP1	2.18E-08	0.68
C12orf34	2.18E-08	1.46
C16orf7	2.18E-08	1.58
TNFAIP3	2.20E-08	0.51
WWC3	2.21E-08	1.48
NFKBIL1	2.23E-08	1.60
NUCB2	2.30E-08	1.51
NSDHL	2.31E-08	1.51
IL1RL1	2.33E-08	0.49
TRIB1	2.35E-08	0.60
TLCD1	2.35E-08	1.50
OSBPL7	2.39E-08	1.50
BTG1	2.48E-08	0.64
CARS	2.50E-08	1.44
HMGCS1	2.52E-08	1.60
MAOA	2.52E-08	0.68
TMEM143	2.54E-08	1.57
ELOVL6	2.55E-08	1.50
CREB3L2	2.55E-08	1.48
FBLN1	2.59E-08	1.46
SNCA	2.61E-08	1.51
NECAB3	2.67E-08	1.49
D2HGDH	2.68E-08	1.55
IRAK2	2.70E-08	0.58
DUSP4	2.71E-08	0.70
SLC16A5	2.74E-08	1.50
FAM206A	2.74E-08	0.69
TTC9C	2.74E-08	0.69
IL11	2.74E-08	0.61
DNAJB9	2.79E-08	1.61
SUV420H1	2.79E-08	0.66
FN1	2.79E-08	0.57
RNPEPL1	2.88E-08	1.42
NEDD4L	2.97E-08	0.69

TSTD2	3.02E-08	0.67
CAMKK2	3.03E-08	1.48
ASB13	3.23E-08	1.45
FOSL1	3.31E-08	0.66
UGDH	3.35E-08	1.53
SEC24C	3.35E-08	1.45
IRS2	3.50E-08	0.62
CCNE2	3.52E-08	1.42
CRYL1	3.55E-08	1.52
TRMT61A	3.55E-08	0.61
CALHM3	3.60E-08	1.84
PCK2	3.60E-08	1.71
FSTL3	3.60E-08	0.67
MFSD3	3.66E-08	1.58
SMAD7	3.68E-08	0.65
BDKRB2	3.72E-08	1.41
CXCL1	3.82E-08	0.55
TOR2A	3.88E-08	1.62
COL13A1	3.98E-08	1.74
BIRC2	4.01E-08	0.66
TIMP4	4.01E-08	1.50
CC2D2A	4.01E-08	0.60
CNFN	4.07E-08	0.67
UPF3B	4.09E-08	0.62
DSCAM	4.13E-08	0.68
PCYT2	4.14E-08	1.47
RNF114	4.22E-08	0.67
UBAP2L	4.35E-08	1.53
TSPYL4	4.35E-08	0.70
CITED2	4.53E-08	0.68
CD274	4.63E-08	0.68
COL5A1	4.80E-08	1.56
RIOK3	4.94E-08	0.66
FLJ22184	5.08E-08	1.57
KLF4	5.10E-08	0.59
LIPE	5.18E-08	1.51
SERF2	5.18E-08	0.65
ACSS2	5.29E-08	1.61
FAM167A	5.44E-08	0.65
KDM4C	5.56E-08	0.69
OR51B5	6.07E-08	1.42

STMN1	6.24E-08	0.68
TCFL5	6.48E-08	1.64
PTPRK	6.48E-08	0.70
HCAR2	6.48E-08	0.63
HCAR3	6.49E-08	0.68
TIPARP	6.49E-08	0.67
TXNL4B	6.50E-08	0.60
AKR1B10	6.59E-08	1.57
ANKRD2	6.74E-08	1.42
BDH1	6.76E-08	1.42
KCNJ15	6.85E-08	0.67
NR3C1	6.93E-08	0.61
ACTR1B	7.19E-08	1.51
MSMO1	7.30E-08	1.46
TMEM129	7.38E-08	1.56
PSIP1	7.77E-08	1.55
GFOD1	7.85E-08	0.52
C20orf201	7.85E-08	1.76
INPP5A	7.86E-08	0.66
WIBG	7.96E-08	1.43
WWP2	8.13E-08	0.62
CALM3	8.55E-08	1.46
USO1	8.64E-08	1.45
JUN	8.66E-08	0.64
KHDRBS1	8.71E-08	1.46
MKRN1	8.78E-08	0.67
A2LD1	8.79E-08	1.47
TAF15	9.01E-08	1.80
AEN	9.01E-08	0.69
KRT6C	9.10E-08	0.67
PPP1R15A	9.24E-08	0.55
LOC728392	9.30E-08	1.65
GDF15	9.65E-08	1.44
RNF222	1.02E-07	1.97
PIR	1.02E-07	1.53
PLS3	1.03E-07	0.70
PLEKHJ1	1.04E-07	1.50
ABTB2	1.04E-07	0.68
HSD17B7	1.04E-07	1.43
AREG	1.04E-07	0.68
TMX4	1.07E-07	1.42

CALY	1.10E-07	1.62
RAB11FIP4	1.12E-07	1.52
C3orf52	1.12E-07	0.70
CERCAM	1.14E-07	1.48
SPRY2	1.14E-07	0.59
FLYWCH1	1.14E-07	1.55
EPPK1	1.14E-07	1.51
CDC42EP5	1.14E-07	1.48
ELL	1.14E-07	0.70
FAM55C	1.15E-07	1.44
SERINC1	1.15E-07	0.67
KCNE1L	1.15E-07	0.64
C6orf62	1.16E-07	1.48
SMG1	1.18E-07	0.67
ZNF488	1.24E-07	1.61
C11orf75	1.26E-07	1.69
KIAA0232	1.28E-07	1.56
EEF2K	1.30E-07	1.45
PER2	1.30E-07	0.69
BCL2L1	1.33E-07	0.64
ASB1	1.35E-07	0.69
HCFC1R1	1.36E-07	1.52
RPP30	1.44E-07	0.70
C14orf1	1.50E-07	1.42
C9orf69	1.50E-07	1.54
MKI67	1.51E-07	1.43
CCDC76	1.52E-07	0.70
SEMA4C	1.52E-07	1.45
SNAI2	1.55E-07	0.59
CEACAM1	1.59E-07	0.64
VSX1	1.59E-07	1.61
KAT7	1.61E-07	0.70
BATF2	1.61E-07	0.69
CSF2	1.62E-07	0.64
AKAP12	1.65E-07	0.69
UPP1	1.65E-07	0.65
MMP1	1.71E-07	0.59
ZC3HAV1L	1.79E-07	1.59
AMD1	1.85E-07	1.45
HCN2	1.87E-07	1.65
DNMT3B	1.87E-07	1.58

MICB	1.88E-07	1.51
LBR	1.97E-07	1.55
ZFC3H1	1.98E-07	1.45
UNKL	1.98E-07	1.73
HGSNAT	2.16E-07	1.48
IDH2	2.17E-07	1.48
LAMTOR2	2.20E-07	1.44
SPATA5L1	2.23E-07	0.70
BAMBI	2.32E-07	0.61
HBEGF	2.33E-07	0.60
NEDD9	2.38E-07	0.64
AKAP8L	2.40E-07	0.67
C12orf4	2.42E-07	0.70
CLMP	2.44E-07	0.68
ABL2	2.44E-07	0.62
AHNAK2	2.47E-07	0.70
FAM120B	2.58E-07	1.43
CDKN2D	2.62E-07	1.46
DOK7	2.62E-07	0.68
CCDC18	2.71E-07	1.56
HIST1H1A	2.75E-07	1.56
MAFB	2.87E-07	0.59
FDPS	2.92E-07	1.62
ERP27	2.93E-07	0.61
IGFLR1	2.93E-07	1.43
CHIC2	3.04E-07	0.63
PNLIPRP3	3.07E-07	0.67
IDI1	3.23E-07	1.49
GATSL3	3.33E-07	1.50
IL1R2	3.35E-07	0.59
ASNS	3.37E-07	1.57
ETS1	3.48E-07	0.68
DAPP1	3.52E-07	0.64
ABCB10	3.53E-07	1.48
NUDT18	3.56E-07	1.43
NDUFC2	3.81E-07	0.66
SLC25A38	3.83E-07	0.70
TREX1	3.91E-07	1.44
TM7SF2	3.92E-07	1.60
LSP1	4.00E-07	1.67
ZZZ3	4.02E-07	0.68

SMG7	4.17E-07	0.70
ANK3	4.31E-07	1.44
E2F1	4.33E-07	1.43
FOXP4	4.47E-07	1.44
MBOAT2	4.50E-07	0.71
ZFAND2A	4.70E-07	0.69
HDAC9	4.78E-07	0.57
RND3	4.78E-07	0.70
NFIC	4.84E-07	1.47
IP6K1	4.87E-07	1.54
CASP10	4.90E-07	0.67
ID1	4.91E-07	1.61
ELK3	4.95E-07	0.65
TPSG1	4.99E-07	1.42
REEP6	5.13E-07	1.45
RSF1	5.13E-07	0.68
LIMK1	5.21E-07	1.42
KPNA4	5.32E-07	0.71
COX19	5.76E-07	0.65
LY9	5.77E-07	1.56
IFITM10	5.95E-07	1.51
C17orf108	6.30E-07	1.70
CREB3L4	6.33E-07	1.51
SFXN5	6.43E-07	1.50
HIVEP3	6.44E-07	1.46
TSC22D1	6.44E-07	0.63
RALGDS	6.82E-07	1.52
GALR3	6.91E-07	1.57
HIST1H1B	6.96E-07	1.69
CD44	7.24E-07	0.67
KCTD19	7.33E-07	1.71
ITGA5	7.54E-07	0.70
NAV3	7.69E-07	0.64
GRHL3	7.80E-07	0.68
OSR2	7.82E-07	0.60
ZFP57	8.10E-07	0.67
IGF2	8.15E-07	0.69
CEBPD	8.16E-07	1.64
EIF4EBP2	8.16E-07	1.46
CYP1A1	8.16E-07	0.61
C19orf60	8.57E-07	1.47

RBM4	8.76E-07	0.69
ADA	9.48E-07	1.49
PRMT5	9.71E-07	0.70
PANK2	9.73E-07	0.69
WIPI1	9.93E-07	1.45
IFFO1	1.02E-06	0.68
RIMBP3	1.03E-06	1.46
C20orf160	1.16E-06	1.61
FBRSL1	1.17E-06	1.47
BTBD7	1.24E-06	0.70
HEXDC	1.26E-06	1.47
CISH	1.27E-06	1.49
AKAP2	1.29E-06	0.67
LRP3	1.31E-06	1.75
GJC2	1.31E-06	1.51
ACCN2	1.33E-06	1.48
CDKN2AIP	1.35E-06	0.70
RAB26	1.40E-06	1.61
TRAPPC6A	1.40E-06	1.48
C1orf116	1.42E-06	0.69
RNF32	1.46E-06	1.62
SMG9	1.46E-06	0.67
ARHGEF3	1.47E-06	0.69
SCG5	1.48E-06	0.65
DOCK4	1.53E-06	0.69
IL6	1.53E-06	0.56
DUSP10	1.59E-06	0.70
DST	1.60E-06	0.70
TMCO4	1.63E-06	1.45
ATF3	1.65E-06	0.66
LYNX1	1.70E-06	1.86
SFXN2	1.73E-06	1.44
SOCS3	1.80E-06	0.62
MAP1S	1.88E-06	1.49
MARCH4	1.93E-06	0.70
ZNF497	1.97E-06	1.79
EPHX2	2.01E-06	1.44
MON1B	2.03E-06	1.67
SMARCA2	2.05E-06	1.47
SDCBP2	2.11E-06	0.68
ARHGAP33	2.18E-06	1.54

KLHL21	2.19E-06	0.71
DIABLO	2.21E-06	1.48
EPHA4	2.24E-06	0.67
SPRR1B	2.31E-06	0.70
NOB1	2.32E-06	0.68
PTX3	2.37E-06	0.67
SYT12	2.56E-06	1.48
SGSM3	2.57E-06	1.42
AKR1B15	2.67E-06	1.43
ITGB2	2.67E-06	1.57
TMC8	2.74E-06	0.65
PEAR1	2.79E-06	0.70
ZCCHC2	2.83E-06	1.45
GATA3	2.86E-06	0.63
DGCR14	2.88E-06	0.70
JUNB	2.90E-06	0.70
CD59	2.95E-06	0.70
KCNQ2	2.98E-06	1.96
SYNGR4	2.98E-06	1.60
HAGHL	3.02E-06	1.42
SGK3	3.03E-06	1.59
TRAK2	3.04E-06	1.51
DNM1	3.27E-06	1.43
PRRG1	3.37E-06	0.63
IL37	3.50E-06	0.65
HIST1H1D	3.52E-06	1.44
SLC37A1	3.90E-06	1.42
LIMA1	4.06E-06	0.70
HMGB2	4.12E-06	1.43
HIST1H2AH	4.38E-06	1.43
PER1	4.38E-06	0.66
DNMBP	4.85E-06	0.66
GDA	4.97E-06	0.70
SERPINB7	5.17E-06	0.70
CD86	5.43E-06	1.81
STEAP4	5.81E-06	0.65
PNPLA2	6.17E-06	1.57
IQGAP3	6.61E-06	1.41
CSF3	6.76E-06	0.68
PI4K2B	7.03E-06	1.49
ACACB	7.88E-06	1.44

PFKL	8.67E-06	1.73
PARVB	8.67E-06	1.43
ZNF350	8.77E-06	0.70
HCN3	8.79E-06	1.47
IBTK	9.32E-06	1.43
EMILIN1	1.01E-05	1.61
AOX1	1.01E-05	0.65
RUSC1-AS1	1.09E-05	1.52
C11orf9	1.15E-05	1.54
RDM1	1.17E-05	1.54
HMG20B	1.30E-05	1.63
PHTF1	1.30E-05	1.44
CRCT1	1.34E-05	0.69
TMEM50B	1.37E-05	1.44
NFAT5	1.37E-05	0.66
ATXN10	1.42E-05	1.44
KLF5	1.43E-05	0.69
LHX3	1.48E-05	1.58
C16orf93	1.50E-05	1.45
NUPR1	1.85E-05	1.45
HMX1	1.92E-05	1.44
VGLL3	1.93E-05	0.61
RSAD2	2.18E-05	0.68
HERC5	2.29E-05	0.49
C1orf27	2.37E-05	0.67
NFKBIZ	2.41E-05	0.63
MOCOS	2.48E-05	1.50
CD70	2.63E-05	1.53
FICD	3.27E-05	1.42
ABCA1	3.27E-05	0.69
HIST1H2AL	3.36E-05	1.45
GPR150	3.70E-05	1.58
TINAGL1	3.98E-05	1.65
SCARF2	4.08E-05	1.48
HUS1B	4.19E-05	0.67
YJEFN3	4.62E-05	1.68
MED10	4.74E-05	0.71
PTMS	4.79E-05	1.44
LRRC45	4.83E-05	1.47
METTL7A	5.13E-05	1.62
RAVER1	5.58E-05	1.51

SBNO2	6.62E-05	1.43
TMEM52	7.14E-05	1.52
PCSK1N	9.27E-05	1.69
RBAK-LOC389458	9.28E-05	1.46
PKD1	9.53E-05	1.48
SPRR2A	1.03E-04	0.67
MEX3D	1.07E-04	1.45
ELFN1	1.09E-04	1.49
RHBDL1	1.18E-04	1.48
PRR25	1.33E-04	1.50
C11orf91	1.42E-04	0.66
KRTAP3-3	1.45E-04	1.51
TGFA	1.53E-04	0.71
ANKRD10	1.67E-04	0.70
RFX8	2.19E-04	1.58
UTS2R	2.44E-04	1.75
UNCX	2.48E-04	1.46
MOB1B	2.62E-04	1.42
PPP1R14A	2.66E-04	1.60
C1orf229	2.98E-04	1.51
ZNF699	3.11E-04	0.70
HOXA10	3.42E-04	1.45
HKR1	3.52E-04	0.68
DPF2	4.63E-04	0.65
TPP1	5.60E-04	1.52
TMEM156	7.79E-04	0.69
CISD3	1.11E-03	2.09
UCP3	1.18E-03	1.78
BHLHE23	1.32E-03	1.54
P2RY1	1.75E-03	1.47
MOCS3	2.39E-03	1.53
GP9	2.58E-03	1.45
C3AR1	3.68E-03	1.81
VAMP2	9.57E-03	1.51
ZNF467	1.07E-02	1.47
SSTR3	1.52E-02	1.77
MTRNR2L10	1.88E-02	1.45
ATXN7L2	2.02E-02	1.42
KLRG2	3.37E-02	1.53
FAM57B	3.72E-02	1.52
EVX1	3.82E-02	1.57

LRRIQ3	4.86E-02	1.44
