# Supplementary material for: Integrative analysis of the microRNA-mRNA response to radiochemotherapy in primary head and neck squamous cell carcinoma cells
Source: BMC Genomics. 2015 Sep 2;16(1):654. doi: 10.1186/s12864-015-1865-x (PMC4557600; doi:10.1186/s12864-015-1865-x)
Supplement: Additional file 7: — Significantly deregulated mRNAs in primary HN2092 after in vitro radiochemotherapy treatment. (PDF 143 kb) [file 12864_2015_1865_MOESM7_ESM.pdf]

Additional file 7 Significantly deregulated mRNAs in HN2092 after radiochemotherapy treatment (adjusted p-value<0.05)

Gene	adjusted p-value	fold-change
RPL28	3.68E-08	0.44
TRMT61A	6.87E-08	0.49
WLS	6.87E-08	0.52
ASNS	6.87E-08	1.91
SLC38A10	6.87E-08	1.98
HEXDC	6.87E-08	2.16
PCK2	6.87E-08	2.25
JDP2	6.87E-08	2.39
HS1BP3	7.11E-08	1.90
ZBTB24	8.78E-08	0.53
C1S	8.78E-08	1.83
IFRD1	1.07E-07	0.48
LIG1	1.22E-07	1.71
SFXN2	1.37E-07	1.67
DHRS1	1.52E-07	1.58
CDC45	1.52E-07	1.66
CPT2	1.52E-07	1.73
NINL	1.52E-07	1.76
CBS	1.52E-07	1.96
KCNE1L	1.65E-07	0.34
RUNX1	1.65E-07	0.57
RPL22L1	1.65E-07	0.61
GFOD1	1.93E-07	0.57
TRIOBP	1.93E-07	1.98
SFI1	1.95E-07	1.79
HSPA1A	2.02E-07	1.59
FUT1	2.02E-07	1.60
PACS2	2.12E-07	1.81
CC2D2A	2.44E-07	0.48
TMEM143	2.44E-07	1.94
RPL3	2.47E-07	1.68
MEGF6	2.60E-07	1.76
EDN1	2.80E-07	0.59
CREB3L4	2.80E-07	1.58
NOTCH3	2.81E-07	1.75
SYTL1	3.01E-07	1.64
LOC100287177	3.09E-07	1.66

CTF1	3.09E-07	1.67
STAG3	3.52E-07	1.53
C8orf33	3.76E-07	0.63
PHGDH	4.36E-07	1.85
CBX6	4.68E-07	1.73
PLD6	5.12E-07	0.61
MYLK2	5.38E-07	0.57
FASN	5.74E-07	1.52
RHBDF2	5.74E-07	1.67
TRADD	5.99E-07	1.61
MSRB2	6.72E-07	1.52
GPT2	6.94E-07	1.51
TROAP	8.06E-07	0.59
HGSNAT	8.06E-07	1.58
PIGZ	8.53E-07	1.66
HES6	8.56E-07	1.53
HMGA2	8.94E-07	0.64
LLGL2	9.67E-07	1.48
SHANK3	9.67E-07	1.50
MVD	9.67E-07	1.69
IDH2	1.00E-06	1.58
ANKRD10	1.01E-06	0.54
SNRPA1	1.01E-06	0.64
PDRG1	1.01E-06	0.67
ALDH1L2	1.01E-06	1.87
OSBPL5	1.01E-06	2.13
HIST1H1D	1.01E-06	2.41
PLAT	1.01E-06	1.55
ZDHHC12	1.08E-06	1.46
ZNF256	1.15E-06	0.65
ZNF672	1.15E-06	1.59
METTL3	1.15E-06	1.62
LEPROT	1.18E-06	0.54
ITPK1	1.18E-06	0.59
ABL2	1.18E-06	0.63
SLC6A15	1.18E-06	0.67
BRF2	1.18E-06	0.70
MKRN1	1.24E-06	0.57
RECQL	1.27E-06	1.46
CLEC16A	1.30E-06	1.52
POLA2	1.30E-06	1.54

NLRP7	1.30E-06	1.59
ZNF502	1.35E-06	0.63
DUSP12	1.36E-06	0.65
TST	1.36E-06	1.83
TDRD9	1.38E-06	1.49
GAA	1.43E-06	1.52
RMI2	1.43E-06	1.73
KLHL29	1.45E-06	1.61
ASF1B	1.49E-06	1.75
ARHGAP27	1.50E-06	0.64
RPL17	1.56E-06	0.64
PDSS2	1.72E-06	0.66
TMEM201	1.72E-06	0.70
PGLS	1.81E-06	1.54
ADCK5	1.81E-06	1.49
SDF2L1	1.82E-06	1.46
TAF9	1.82E-06	0.69
HKDC1	1.84E-06	1.52
CYCS	1.99E-06	0.68
SLMO1	1.99E-06	0.70
PARVB	2.01E-06	1.53
PGAP3	2.07E-06	0.66
EPHX2	2.16E-06	1.71
IRF5	2.16E-06	1.44
CTH	2.16E-06	1.73
NRP2	2.20E-06	0.64
ZZZ3	2.20E-06	0.68
NOXA1	2.20E-06	1.73
PPP1R35	2.37E-06	1.50
MLH1	2.53E-06	1.63
CYR61	2.63E-06	0.66
ADM	2.72E-06	0.60
CRELD2	2.72E-06	1.66
HABP4	2.73E-06	1.49
UBE2C	2.76E-06	0.70
SGK1	2.79E-06	0.61
MBIP	2.98E-06	0.63
PER1	2.98E-06	0.69
PTPN18	3.00E-06	1.49
TMEM129	3.02E-06	1.61
SETD3	3.07E-06	0.70

REEP6	3.07E-06	1.61
PRRT3	3.15E-06	1.51
SLC6A9	3.22E-06	1.62
HIST2H2AC	3.27E-06	1.87
DNMT3B	3.38E-06	1.57
TRMU	3.44E-06	0.65
RNF213	3.44E-06	1.47
HKR1	3.49E-06	0.65
SPTLC2	3.49E-06	2.12
PPOX	3.54E-06	1.44
C17orf90	3.54E-06	1.44
C1orf56	3.58E-06	1.42
EVL	3.60E-06	1.52
GIT2	3.60E-06	1.54
POLA1	3.64E-06	1.54
KIAA1432	3.90E-06	0.58
CREG2	3.92E-06	1.58
CDK2AP2	4.28E-06	1.42
HEATR7A	4.28E-06	1.51
IGFLR1	4.30E-06	1.62
ARHGEF9	4.34E-06	1.43
MTR	4.34E-06	1.44
SPATA5L1	4.42E-06	0.70
SERF2	4.45E-06	0.66
UCN2	4.82E-06	0.60
C5orf45	4.82E-06	1.42
PRR4	4.88E-06	0.67
C11orf75	4.88E-06	1.56
TXNL4B	5.04E-06	0.61
CABLES2	5.05E-06	1.59
ACTR1B	5.08E-06	1.61
ZNF614	5.09E-06	0.61
SFXN5	5.12E-06	1.45
NUP210	5.12E-06	1.55
KCNG1	5.14E-06	1.73
NLGN4Y	5.21E-06	0.64
C1orf51	5.22E-06	0.68
DLG3	5.22E-06	1.44
C1orf55	5.23E-06	0.65
MAP3K9	5.29E-06	0.58
PLEKHJ1	5.29E-06	1.66

SH2D3C	5.32E-06	1.52
UBOX5	5.46E-06	0.71
ZNF775	5.46E-06	1.52
C8orf40	5.75E-06	1.42
ZNF362	5.80E-06	1.53
HSD17B7	6.04E-06	1.50
ZNF750	6.29E-06	0.60
ARL8B	6.29E-06	0.65
SETDB1	6.29E-06	0.70
COMTD1	6.29E-06	1.52
CALHM3	6.50E-06	1.42
PIP5KL1	6.51E-06	1.46
SPRY2	6.54E-06	0.68
NCAPH2	6.60E-06	0.68
RGS16	6.84E-06	0.60
BCCIP	6.84E-06	0.66
GINS1	6.84E-06	1.44
ZNF789	6.84E-06	1.58
SLC7A11	6.94E-06	1.62
ARMCX2	7.03E-06	1.48
C16orf72	7.11E-06	0.67
ACOX3	7.19E-06	1.45
BRD4	7.23E-06	0.63
ESPL1	7.23E-06	1.49
NECAB3	7.31E-06	1.43
SLC35F2	7.37E-06	0.67
FLVCR1	7.37E-06	0.69
IFFO1	7.84E-06	0.69
DOCK9	7.90E-06	0.66
B3GNT3	7.90E-06	1.48
NT5C3	7.91E-06	0.67
ZNF720	8.40E-06	1.86
DAPP1	8.49E-06	0.66
HIST1H4D	8.51E-06	1.98
GET4	8.54E-06	0.70
HIST1H1A	8.88E-06	3.50
STAT2	9.08E-06	1.55
ZSCAN5A	9.17E-06	0.68
FXYD3	9.22E-06	0.61
LEPR	9.23E-06	0.58
RPL27A	9.23E-06	0.64

LOXL1	9.23E-06	1.47
C2orf43	9.38E-06	0.64
GLS	9.38E-06	0.67
PER2	9.54E-06	0.70
HEATR5A	9.54E-06	1.46
C9orf40	9.73E-06	1.47
AGRN	9.76E-06	1.65
HNRPDL	9.79E-06	0.57
TMSB15B	9.79E-06	1.50
SEPT10	9.79E-06	1.54
FEM1B	1.03E-05	0.68
ARHGEF17	1.03E-05	1.45
C15orf52	1.03E-05	1.52
GNAZ	1.07E-05	1.42
SPG11	1.07E-05	1.43
CHAC1	1.07E-05	1.62
DNAJC2	1.09E-05	0.70
HSD17B8	1.09E-05	1.51
ST6GALNAC2	1.09E-05	1.64
FXC1	1.10E-05	0.71
PRSS16	1.16E-05	1.51
EGFR	1.18E-05	0.70
KANK2	1.18E-05	1.42
APCDD1	1.18E-05	1.46
SHMT2	1.18E-05	1.50
UPF3B	1.21E-05	0.67
TFB2M	1.22E-05	0.62
OXCT1	1.27E-05	1.60
NAT8L	1.31E-05	0.69
MIPEP	1.32E-05	1.43
ATG16L2	1.34E-05	1.49
CERCAM	1.40E-05	1.53
IQGAP3	1.40E-05	1.53
MEGF8	1.41E-05	1.73
C2orf55	1.42E-05	1.69
KIAA0101	1.48E-05	1.46
HCAR3	1.49E-05	0.69
MFAP3	1.50E-05	0.68
KLHL22	1.52E-05	1.46
PSMG4	1.53E-05	1.52
AHNAK	1.55E-05	0.70

WHAMM	1.59E-05	0.70
ABCB6	1.59E-05	1.47
EEF2K	1.62E-05	1.60
MBLAC2	1.62E-05	1.60
ZNF777	1.66E-05	0.70
UBFD1	1.69E-05	0.65
CWC27	1.69E-05	0.69
OVGP1	1.70E-05	0.70
ACCS	1.71E-05	1.50
C5orf42	1.75E-05	1.52
C8orf55	1.76E-05	1.47
ACSS2	1.77E-05	1.55
ANKRD35	1.78E-05	1.54
HOOK2	1.80E-05	1.43
ACOT11	1.80E-05	1.44
HAGH	1.80E-05	1.49
RECQL5	1.84E-05	0.69
GARS	2.03E-05	1.49
DOK1	2.03E-05	1.57
DTNBP1	2.03E-05	1.48
HIST1H4L	2.13E-05	1.90
NAGK	2.16E-05	1.44
HIST1H1B	2.26E-05	2.99
PLK2	2.27E-05	0.68
C7orf53	2.32E-05	0.58
FAM126A	2.33E-05	0.65
RNF114	2.33E-05	0.70
TMSB15A	2.33E-05	1.42
C1orf226	2.33E-05	1.48
THUMPD2	2.44E-05	0.61
N4BP2L2	2.46E-05	0.64
KCNRG	2.48E-05	0.70
BACE1	2.50E-05	1.65
LSS	2.54E-05	1.49
CLEC2B	2.54E-05	0.66
RGS2	2.60E-05	0.56
ANKRD2	2.60E-05	1.50
NDUFC2	2.61E-05	0.61
ZNF837	2.70E-05	1.56
G2E3	2.71E-05	0.59
PLK3	2.71E-05	0.69

ALDH4A1	2.75E-05	1.51
TCP1	2.76E-05	0.63
TET1	2.78E-05	1.58
ACSF3	2.78E-05	1.45
FLG	2.80E-05	0.63
ADM2	2.97E-05	1.54
HSPG2	2.97E-05	1.67
PLAU	3.00E-05	0.70
LHPP	3.04E-05	1.44
STMN1	3.19E-05	0.68
C18orf21	3.20E-05	0.67
ARL6IP1	3.30E-05	0.64
CCR10	3.31E-05	1.42
EVI5L	3.31E-05	1.54
CCDC88C	3.32E-05	0.70
B9D1	3.32E-05	1.46
C17orf109	3.36E-05	0.65
TUBB3	3.44E-05	1.45
C1orf109	3.47E-05	0.64
ZNF354B	3.47E-05	0.69
WDR3	3.56E-05	0.68
AHSA2	3.67E-05	1.60
ZNF317	3.68E-05	0.68
SAP25	3.72E-05	1.45
ELF3	3.82E-05	0.64
RPAP2	3.92E-05	0.70
CNOT1	4.03E-05	0.69
MSI2	4.03E-05	0.65
SERPINB2	4.03E-05	0.63
C17orf108	4.14E-05	1.61
ACY1	4.15E-05	1.44
MANF	4.16E-05	1.74
HERPUD1	4.27E-05	1.59
PTGER4	4.28E-05	0.61
SEPT6	4.31E-05	1.46
SLC19A1	4.34E-05	0.68
HIST1H1E	4.34E-05	2.18
GNGT1	4.37E-05	0.67
CCDC92	4.40E-05	1.47
PRH2	4.41E-05	0.67
HMGCS1	4.42E-05	1.59

DHRS2	4.52E-05	1.43
MICALCL	4.73E-05	1.46
PCSK9	4.95E-05	1.52
RSPH3	5.19E-05	1.46
HIST1H2AJ	5.23E-05	1.94
PSAT1	5.45E-05	1.54
HIST1H2AL	5.51E-05	2.65
NUDT8	5.83E-05	1.47
CAT	5.94E-05	1.80
OVOL1	6.10E-05	0.64
FDPS	6.15E-05	1.45
D2HGDH	6.18E-05	1.47
HIST1H2AB	6.21E-05	1.54
EEFSEC	6.34E-05	1.50
C3orf52	6.43E-05	0.69
CD55	6.52E-05	0.70
PDE5A	6.54E-05	1.46
CBY1	6.71E-05	1.46
ATHL1	6.93E-05	1.45
NEK1	7.08E-05	1.47
ANKRD36B	7.08E-05	1.59
GRHL3	7.10E-05	0.68
RDM1	7.25E-05	1.45
DDX10	7.29E-05	0.68
BCAR3	7.30E-05	0.69
DHFR	7.38E-05	1.46
STK17B	7.41E-05	0.70
GDF15	7.46E-05	1.45
AARS	7.54E-05	1.50
ANKRD36	7.68E-05	1.70
SLX1A	8.24E-05	1.46
HSPA5	8.53E-05	2.02
TMEM187	8.56E-05	1.59
KATNAL1	8.56E-05	1.57
SMNDC1	8.66E-05	0.62
ASL	8.82E-05	1.69
MLXIP	8.89E-05	0.67
MMD	8.91E-05	1.58
ACAD11	8.98E-05	1.42
HIST1H2AK	9.12E-05	1.93
IBA57	9.32E-05	0.63

HIST1H2AI	9.37E-05	1.94
HSBP1L1	9.39E-05	0.69
CARS	9.56E-05	1.93
ULBP1	1.01E-04	1.42
S100PBP	1.03E-04	1.43
PSKH1	1.04E-04	1.85
FOSB	1.04E-04	0.67
C21orf91	1.12E-04	0.66
HIST1H2AG	1.14E-04	3.10
IFIT2	1.15E-04	0.60
RPS6KA2	1.17E-04	1.87
PRODH	1.17E-04	1.43
ATAT1	1.21E-04	1.53
PDCD6IP	1.23E-04	1.43
IRS2	1.27E-04	0.66
GEMIN2	1.27E-04	0.68
LYSMD2	1.27E-04	0.69
SLC30A1	1.33E-04	0.69
MICB	1.36E-04	1.45
RABL2A	1.36E-04	1.49
NIPSNAP3A	1.40E-04	1.46
MMP28	1.43E-04	1.48
SLC13A3	1.43E-04	1.48
ASS1	1.45E-04	1.44
BLOC1S3	1.48E-04	0.71
B4GALNT1	1.48E-04	1.43
CCDC50	1.50E-04	0.70
PASK	1.52E-04	1.46
CSNK1A1L	1.55E-04	0.70
HIST1H2AH	1.67E-04	2.11
ITFG2	1.71E-04	0.70
ACADM	1.76E-04	1.44
CHP2	1.76E-04	1.63
CLSPN	1.76E-04	1.72
OASL	1.79E-04	0.57
TIMM13	1.82E-04	0.68
FAT1	1.95E-04	0.63
USP53	1.99E-04	0.69
HSP90B1	2.03E-04	2.01
PIDD	2.09E-04	1.42
HIST1H2BJ	2.13E-04	1.44

ATF3	2.17E-04	0.66
SEL1L3	2.20E-04	1.43
CCDC18	2.32E-04	1.49
EIF4A2	2.32E-04	0.67
VEGFA	2.38E-04	1.61
CTHRC1	2.40E-04	1.47
AVPR1A	2.43E-04	0.65
CBR4	2.44E-04	1.45
CCNE2	2.45E-04	1.58
EXPH5	2.49E-04	0.69
TMEM107	2.50E-04	1.50
NDUFA7	2.57E-04	1.43
INSIG1	2.58E-04	1.61
CALB1	2.69E-04	0.65
YBX2	2.69E-04	1.42
DNMT3A	2.82E-04	1.46
ASB7	2.84E-04	0.63
KRR1	2.85E-04	0.65
HIST1H2AM	3.03E-04	1.73
FRS2	3.06E-04	0.66
EFNB2	3.11E-04	0.68
TMX4	3.42E-04	1.45
C18orf56	3.54E-04	1.55
TCFL5	3.58E-04	1.48
KIAA0513	3.61E-04	1.45
ID1	3.72E-04	1.44
EGR4	3.80E-04	0.69
HIST1H4E	3.89E-04	2.21
CHM	3.95E-04	0.63
NRAS	3.96E-04	0.67
HIST1H4A	3.96E-04	1.86
CRELD1	4.10E-04	1.56
SHMT1	4.15E-04	1.60
HIST1H2BB	4.31E-04	1.41
ANK1	4.42E-04	1.44
HIST1H2BD	4.52E-04	1.44
GBP5	4.54E-04	1.42
ZNF426	4.64E-04	0.71
POLR1C	4.67E-04	0.68
MED10	4.80E-04	0.67
FZR1	5.04E-04	0.71

C11orf82	5.06E-04	0.71
CHODL	5.06E-04	0.67
STX12	5.06E-04	1.45
HIST1H2AD	5.11E-04	1.46
RABGGTB	5.40E-04	0.68
ACACB	5.42E-04	1.62
HIST1H4C	5.42E-04	1.55
HIST1H3D	5.55E-04	1.95
MRPL44	5.83E-04	0.69
TOR2A	5.84E-04	1.42
RND3	5.88E-04	0.52
EFNA1	6.19E-04	0.68
ENC1	6.28E-04	0.61
CNFN	6.57E-04	0.60
ZFC3H1	6.72E-04	1.44
HIST1H3B	6.83E-04	1.83
HIST1H3H	6.96E-04	1.86
CCNB1	7.25E-04	0.68
FOSL1	7.59E-04	0.71
AEN	7.77E-04	0.70
HIST1H4K	7.94E-04	1.94
MOCOS	8.01E-04	1.46
RRN3	8.05E-04	0.67
PMS1	8.12E-04	1.43
MCMBP	8.43E-04	0.68
HIST1H4I	8.58E-04	1.89
DCBLD1	9.27E-04	1.64
FAM111B	9.51E-04	1.66
CT45A1	9.57E-04	0.68
RNF39	9.70E-04	0.65
TMEM63C	9.72E-04	1.47
IKZF5	9.84E-04	0.69
CAPRIN1	9.85E-04	0.70
CXCR7	1.06E-03	0.68
SLC25A33	1.16E-03	0.66
HIST1H3I	1.17E-03	1.81
C9orf3	1.21E-03	0.71
RBM17	1.22E-03	0.69
METTL21D	1.23E-03	0.61
RNF222	1.32E-03	0.68
PHF20L1	1.40E-03	0.70

MDM4	1.52E-03	0.71
HOXB9	1.52E-03	1.41
HIST1H3G	1.58E-03	1.55
MRE11A	1.58E-03	1.52
GADD45A	1.65E-03	0.70
RIOK3	1.77E-03	0.70
PIGX	1.80E-03	1.56
C1orf52	1.80E-03	0.66
TMEM175	1.95E-03	1.42
SOX9	1.97E-03	0.67
SGK3	2.02E-03	1.45
GNAQ	2.08E-03	0.70
HSPA8	2.14E-03	1.43
HIST2H2AB	2.24E-03	1.68
EHF	2.28E-03	0.70
NR2F1	2.30E-03	1.45
HECTD3	2.38E-03	1.50
HIST1H3F	2.39E-03	1.84
HIST1H2AC	2.41E-03	2.11
HIST1H2BF	2.54E-03	2.11
PTPRZ1	2.55E-03	0.69
UBAP2	2.65E-03	1.47
MBTPS2	2.81E-03	0.70
HIST1H4J	3.04E-03	1.89
MEF2BNB	3.13E-03	0.66
SIK1	3.13E-03	0.70
TTYH3	3.17E-03	1.47
EIF4G3	3.32E-03	1.42
C11orf9	3.38E-03	1.45
PSIP1	3.45E-03	1.47
HIST1H4H	3.48E-03	1.81
HIST2H4B	3.60E-03	1.82
HIST1H2BN	3.69E-03	1.42
PRDM1	4.12E-03	0.70
PKD1	4.22E-03	1.51
NR1D2	4.29E-03	0.69
PGD	4.30E-03	1.44
HIST1H4F	4.44E-03	1.83
EWSR1	4.54E-03	0.69
HIST1H2BE	4.71E-03	1.88
NFATC2IP	4.89E-03	1.58

SPARCL1	4.94E-03	0.63
TINAGL1	5.07E-03	1.63
IMMT	5.28E-03	1.62
RBMS1	5.45E-03	0.70
FAM135A	5.79E-03	0.69
RRM2	5.82E-03	1.60
PIM1	6.50E-03	0.65
STIL	6.53E-03	1.49
KLRG2	6.66E-03	1.44
DNAJC10	6.89E-03	1.43
MRGPRG	7.04E-03	1.43
HIST1H4B	7.51E-03	1.77
PINX1	7.55E-03	1.47
HELLS	7.99E-03	1.43
SPRR2B	8.14E-03	0.69
PBX3	8.14E-03	1.49
BRD3	8.90E-03	1.51
C12orf5	9.47E-03	0.70
TTC7A	1.05E-02	1.43
CLK1	1.12E-02	0.66
CD86	1.12E-02	1.50
MYOF	1.18E-02	1.52
MAP1S	1.26E-02	1.64
ABCB10	1.35E-02	1.42
HERC2	1.43E-02	1.61
NRIP1	1.49E-02	0.68
MOCS3	1.59E-02	1.47
TAF15	1.63E-02	1.61
HMG20B	1.72E-02	1.43
SMC1A	1.82E-02	1.56
ZHX3	1.85E-02	1.66
CYP17A1	1.88E-02	1.43
SNAI2	1.95E-02	0.64
MKI67IP	2.03E-02	0.68
CCDC59	2.17E-02	0.70
HIST1H3J	2.17E-02	1.55
HSPA1B	2.19E-02	1.93
PFKL	2.27E-02	1.75
ZC3HAV1L	2.36E-02	1.56
ARPC1B	2.49E-02	1.48
FBRSL1	2.53E-02	1.74

RBAK-LOC389458	2.58E-02	1.49
ZKSCAN1	2.66E-02	1.44
EPPK1	2.91E-02	2.11
TXNIP	2.93E-02	0.66
UCP3	2.98E-02	1.77
EMILIN1	2.98E-02	1.82
UNCX	3.41E-02	1.70
TMEM167B	3.46E-02	0.64
VAMP2	3.47E-02	1.80
HAPLN2	3.52E-02	2.07
MEX3D	3.59E-02	1.46
PARP1	3.59E-02	1.42
CISD3	3.61E-02	2.06
PPP1R14A	3.76E-02	1.62
SMG5	4.04E-02	0.67
TMEM95	4.11E-02	2.21
IQSEC3	4.14E-02	1.59
KCTD19	4.16E-02	1.55
C20orf201	4.19E-02	1.77
PNPLA2	4.20E-02	1.59
HIST4H4	4.20E-02	1.54
ZNF497	4.34E-02	1.75
TPP1	4.39E-02	1.59
DUX4L4	4.56E-02	1.76
COPG	4.73E-02	1.49
DUX4	4.75E-02	1.74
RAVER1	4.94E-02	1.76
ELFN1	4.96E-02	1.67
