# Supplementary material for: Integrative analysis of the microRNA-mRNA response to radiochemotherapy in primary head and neck squamous cell carcinoma cells
Source: BMC Genomics. 2015 Sep 2;16(1):654. doi: 10.1186/s12864-015-1865-x (PMC4557600; doi:10.1186/s12864-015-1865-x)
Supplement: Additional file 8: — Pathway enrichment analysis of differentially expressed genes in HN1957 after radiochemotherapy treatment. (PDF 66 kb) [file 12864_2015_1865_MOESM8_ESM.pdf]

Additional file 8 Pathway enrichment analysis of differentially expressed genes in HN1957 after radiochemotherapy treatment (FDR<0.05)

Gene Set	Number of Proteins in GeneSet	Proteins from Network	p-value	FDR	Nodes
Direct p53 effectors(N)	133	12	0	<1.250E-04	PMAIP1,SERPINE1,JUN,HSPA1A,BCL2L1,ATF3,E2F1,E2F2,GADD45A,CASP10,GDF15,IGFBP3
Small cell lung cancer(K)	86	10	0	<1.429E-04	PTGS2,BCL2L1,LAMB3,FN1,LAMA3,E2F1,E2F2,CCNE2,BIRC3,BIRC2
Regulation of Cholesterol Biosynthesis by SREBP (SREBF)(R)	50	9	0	<1.667E-04	HMGCR,INSIG1,MVK,MVD,HMGCS1,FDPS,SEC24C,SEC24D,DHCR7
Validated transcriptional targets of AP1 family members Fra1 and Fra2(N)	36	8	0	<2.000E-04	MMP1,JUNB,PLAUR,JUN,LAMA3,PLAU,FOSL1,IL6
AP-1 transcription factor network(N)	70	11	0	<2.500E-04	EDN1,MMP1,JUNB,JUN,ATF3,ETS1,PLAU,FOSL1,CSF2,NR3C1,IL6
TNF signaling pathway(K)	110	14	0	<3.333E-04	PTGS2,EDN1,CREB3L4,SOCS3,JUNB,VEGFC,JUN,TNFAIP3,CXCL1,CSF2,IL6,BIRC3,BIRC2,CASP10
ATF-2 transcription factor network(N)	58	11	0	<5.000E-04	JDP2,SOCS3,JUNB,DDIT3,JUN,BCL2L1,IL23A,ATF3,PLAU,GADD45A,IL6
Cholesterol biosynthesis(R)	19	9	0	<1.000E-03	HMGCR,MVK,MVD,HMGCS1,MSMO1,FDPS,DHCR7,HSD17B7,NSDHL
Pathways in cancer(K)	327	19	0	1.11E-04	PTGS2,MMP1,VEGFC,JUN,FGFR3,BCL2L1,LAMB3,RUNX1,FN1,BMP2,LAMA3,ETS1,E2F1,E2F2,CCNE2,IL6,BIRC3,BIRC2,HSP90B1
TGF-beta signaling pathway(P)	80	9	0	1.82E-04	CITED2,JUNB,JUN,BMP2,SMAD7,FOSL1,INHBA,GDF15,BAMBI
Glucocorticoid receptor regulatory network(N)	77	9	0	2.00E-04	MMP1,GATA3,JUN,SMARCC2,SGK1,CSF2,NR3C1,IL6,PCK2
C-MYB transcription factor network(N)	82	9	0	3.33E-04	PTGS2,GATA3,SMARCA2,ADA,ETS1,ETS2,HSPA8,CEBPD,BIRC3
Nucleotide-binding domain, leucine rich repeat containing	47	7	0	6.00E-04	TNFAIP3,BCL2L1,IRAK2,NLRP3,TXNIP,BIRC3,BIRC2

receptor (NLR) signaling pathways(R)					
IL6-mediated signaling events(N)	47	7	0	6.00E-04	SOCS3,JUNB,JUN,BCL2L1,IL6,CEBPD,HSP90B1
Calcineurin-regulated NFAT-dependent transcription in lymphocytes(N)	46	7	0	6.15E-04	PTGS2,GATA3,JUNB,JUN,E2F1,FOSL1,CSF2
Steroid biosynthesis(K)	20	5	0	1.00E-03	MSMO1,CYP27B1,DHCR7,HSD17B7,NSDHL
Amoebiasis(K)	109	10	0	1.06E-03	IL1R2,ITGB2,LAMB3,FN1,LAMA3,SERPINB2,CXCL1,CSF2,IL6,COL5A1
Terpenoid backbone biosynthesis(K)	21	5	0	1.17E-03	HMGCR,MVK,MVD,HMGCS1,FDPS
Dissolution of Fibrin Clot(R)	11	4	0.0001	1.37E-03	SERPINE1,PLAUR,PLAU,SERPINB2
NOD-like receptor signaling pathway(K)	57	7	0.0001	1.70E-03	TNFAIP3,NLRP3,CXCL1,IL6,BIRC3,BIRC2,HSP90B1
HTLV-I infection(K)	260	15	0.0001	1.70E-03	JUN,IL1R2,ITGB2,BCL2L1,CALR,FDPS,ATF3,ETS1,ETS2,E2F1,E2F2,FOSL1,CSF2,XBP1,IL6
Extracellular matrix organization(R)	263	15	0.0001	1.77E-03	MMP1,CD44,SERPINE1,DST,ITGB2,LAMB3,FN1,BMP2,LAMA3,ITGA5,LEPREL2,COL13A1,HSPG2,COL5A1,FBLN1
Regulation of nuclear SMAD2/3 signaling(N)	77	8	0.0001	1.81E-03	GATA3,SERPINE1,JUN,RUNX1,NKX2-5,SMAD7,NR3C1,HSPA8
Urokinase-type plasminogen activator (uPA) and uPAR-mediated signaling(N)	42	6	0.0001	2.32E-03	SERPINE1,PLAUR,ITGB2,FN1,ITGA5,PLAU
Calcium signaling in the CD4+ TCR pathway(N)	26	5	0.0001	2.38E-03	PTGS2,JUNB,JUN,FOSL1,CSF2
Validated transcriptional targets of deltaNp63 isoforms(N)	44	6	0.0002	2.73E-03	IL1A,ADA,RUNX1,CEBPD,COL5A1,IGFBP3
HIF-1-alpha transcription factor network(N)	66	7	0.0002	3.24E-03	EDN1,CITED2,SERPINE1,JUN,ITGB2,ETS1,PFKL
Beta1 integrin cell surface interactions(N)	66	7	0.0002	3.24E-03	PLAUR,LAMB3,FN1,LAMA3,ITGA5,PLAU,COL5A1

Rheumatoid arthritis(K)	90	8	0.0002	3.42E-03	MMP1,IL1A,JUN,ITGB2,IL23A,CXCL1,CSF2,IL6
Oncogene Induced Senescence(R)	30	5	0.0002	3.43E-03	ETS1,ETS2,E2F1,E2F2,ID1
CD40/CD40L signaling(N)	29	5	0.0002	3.44E-03	JUN,TNFAIP3,BCL2L1,BIRC3,BIRC2
E2F transcription factor network(N)	68	7	0.0002	3.50E-03	SERPINE1,SMARCA2,PLAU,E2F1,E2F2,CCNE2,PRMT5
NF-kappa B signaling pathway(K)	91	8	0.0002	3.67E-03	PTGS2,TNFAIP3,BCL2L1,DDX58,PLAU,GADD45B,BIRC3,BIRC2
Validated transcriptional targets of TAp63 isoforms(N)	49	6	0.0003	3.71E-03	PMAIP1,DST,ADA,GADD45A,GDF15,IGFBP3
mets effect on macrophage differentiation(B)	18	4	0.0003	4.20E-03	JUN,ETS1,ETS2,E2F1
Caspase cascade in apoptosis(N)	52	6	0.0004	4.64E-03	LMNB1,DIABLO,LIMK1,BIRC3,BIRC2,CASP10
Transcriptional misregulation in cancer(K)	179	11	0.0004	4.84E-03	DDIT3,IL1R2,BCL2L1,RUNX1,PLAU,HMGA2,CSF2,PER2,IL6,BIRC3,IGFBP3
Unfolded Protein Response(R)	74	7	0.0004	4.92E-03	DDIT3,CALR,ATF3,TPP1,ASNS,XBP1,HSP90B1
Pertussis(K)	75	7	0.0004	4.97E-03	IL1A,JUN,ITGB2,IL23A,ITGA5,NLRP3,IL6
IL23-mediated signaling events(N)	35	5	0.0004	5.05E-03	SOCS3,IL24,IL23A,CXCL1,IL6
Plasminogen activating cascade(P)	9	3	0.0006	7.10E-03	MMP1,PLAUR,SERPINB2
DNA Damage/Telomere Stress Induced Senescence(R)	58	6	0.0007	7.71E-03	LMNB1,CCNE2,HIST1H1D,HIST1H1B,HIST1H1A,HMGA2
BMP receptor signaling(N)	41	5	0.0009	1.01E-02	NOG,BMP2,SMAD7,PPP1R15A,BAMBI
ECM-receptor interaction(K)	86	7	0.0009	1.06E-02	CD44,LAMB3,FN1,LAMA3,ITGA5,HSPG2,COL5A1
Interferon alpha/beta signaling(R)	63	6	0.001	1.10E-02	ISG15,SOCS3,OASL,IFIT3,IFIT2,IFIT1
Hematopoietic cell lineage(K)	88	7	0.0011	1.18E-02	CD44,IL1A,IL1R2,ITGA5,CD59,CSF2,IL6

Hepatitis B(K)	146	9	0.0013	1.35E-02	CREB3L4,JUN,DDX58,E2F1,E2F2,CCNE2,IL6,HSPG2,CASP10
Influenza A(K)	176	10	0.0013	1.38E-02	IL1A,SOCS3,JUN,HSPA1A,FDPS,DDX58,NLRP3,RSAD2,HSPA8,IL6
PI3K-Akt signaling pathway(K)	346	15	0.0014	1.40E-02	CREB3L4,VEGFC,FGFR3,BCL2L1,LAMB3,FN1,LAMA3,ITGA5,CCNE2, SGK1,SGK3,IL6,PCK2,COL5A1,HSP90B1
FGF signaling pathway(N)	46	5	0.0015	1.44E-02	PLAUR,JUN,FGFR3,PLAU,SPRY2
p53 signaling pathway(K)	68	6	0.0015	1.45E-02	PMAIP1,SERPINE1,CCNE2,GADD45B,GADD45A,IGFBP3
role of mitochondria in apoptotic signaling(B)	13	3	0.0017	1.58E-02	BCL2L1,DIABLO,BIRC3
Signaling by TGF-beta Receptor Complex(R)	70	6	0.0017	1.61E-02	SERPINE1,JUNB,SMAD7,PPP1R15A,NEDD4L,BAMBI
Apoptotic execution phase(R)	52	5	0.0025	2.33E-02	LMNB1,HIST1H1D,HIST1H1B,HIST1H1A,BIRC2
Cytokine-cytokine receptor interaction(K)	265	12	0.0029	2.70E-02	IL1A,IL24,VEGFC,IL1R2,CD70,IL23A,BMP2,INHBA,CXCL1,CSF2,IL6,T NFSF9
Protein processing in endoplasmic reticulum(K)	167	9	0.0032	2.80E-02	DDIT3,HSPA1A,CALR,SEC24C,SEC24D,PPP1R15A,XBP1,HSPA8,HSP 90B1
TGF-beta signaling pathway(K)	80	6	0.0033	2.80E-02	NOG,BMP2,SMAD7,INHBA,ID1,BAMBI
Legionellosis(K)	55	5	0.0032	2.84E-02	ITGB2,HSPA1A,CXCL1,HSPA8,IL6
Beta5 beta6 beta7 and beta8 integrin cell surface interactions(N)	17	3	0.0036	3.10E-02	PLAUR,FN1,PLAU
signal transduction through il1r(B)	35	4	0.0037	3.16E-02	IL1A,JUN,IRAK2,IL6
Focal adhesion(K)	206	10	0.004	3.43E-02	VEGFC,JUN,LAMB3,FN1,LAMA3,ITGA5,PARVB,BIRC3,BIRC2,COL5A1
gata3 participate in activating the th2 cytokine genes expression(B)	18	3	0.0042	3.56E-02	GATA3,IL1A,JUNB
nfat and hypertrophy of the heart (B)	37	4	0.0045	3.75E-02	EDN1,NKX2-5,HDAC9,HBEGF
Apoptosis(K)	86	6	0.0046	3.78E-02	IL1A,BCL2L1,IRAK2,BIRC3,BIRC2,CASP10

Bladder cancer(K)	38	4	0.0049	3.89E-02	MMP1,FGFR3,E2F1,E2F2
Intrinsic Pathway for Apoptosis(R)	38	4	0.0049	3.89E-02	PMAIP1,BCL2L1,DIABLO,E2F1
Parkinson disease(P)	61	5	0.0049	3.99E-02	SNCA,HSPA1A,CCNE2,TOR2A,HSPA8
Regulation of retinoblastoma protein(N)	62	5	0.0053	4.05E-02	JUN,E2F1,E2F2,CSF2,CEBPD
Vitamin B6 metabolism(K)	6	2	0.0053	4.11E-02	AOX1,PSAT1
Toxoplasmosis(K)	119	7	0.0057	4.30E-02	HSPA1A,BCL2L1,LAMB3,LAMA3,HSPA8,BIRC3,BIRC2
Regulation of Insulin-like Growth Factor (IGF) Transport and Uptake by Insulin-like Growth Factor Binding Proteins (IGFBPs)(R)	21	3	0.0065	4.89E-02	MMP1,IGF2,IGFBP3

(B) BioCarta, (K) KEGG Pathway, (N) NCI - Nature Curated Data, (P) pantherdb, (R) Reactome
