# Supplementary material for: Integrative analysis of the microRNA-mRNA response to radiochemotherapy in primary head and neck squamous cell carcinoma cells
Source: BMC Genomics. 2015 Sep 2;16(1):654. doi: 10.1186/s12864-015-1865-x (PMC4557600; doi:10.1186/s12864-015-1865-x)
Supplement: Additional file 9: — Pathway enrichment analysis of differentially expressed genes in HN2092 after radiochemotherapy treatment. (PDF 44 kb) [file 12864_2015_1865_MOESM9_ESM.pdf]

Additional file 9 Pathway enrichment analysis of differentially expressed genes in HN2092 after radiochemotherapy treatment (FDR<0.05)

Gene Set	Number of Proteins in GeneSet	Proteins from Network	p-value	FDR	Nodes
RNA Polymerase I, RNA Polymerase III, and Mitochondrial Transcription(R)	109	13	0	<1.000E-04	HIST1H2BN,HIST1H2BJ,HIST1H4A,RRN3,HIST2H2AC,BRF2,POLR1C,HIST1H2AB,HIST1H2AC,HIST1H2AD,HIST1H2BB,HIST1H2BD,HIST1H2AJ
Mitotic Prophase(R)	99	13	0	<1.111E-04	HIST1H2BN,HIST1H2BJ,HIST1H4A,HIST2H2AC,NCAPH2,HIST1H2AB,HIST1H2AC,HIST1H2AD,NUP210,HIST1H2BB,HIST1H2BD,CCNB1,HIST1H2AJ
Alcoholism(K)	180	17	0	<1.250E-04	HIST1H2BN,HIST1H2BJ,CREB3L4,HIST1H4A,GNGT1,HIST2H2AB,HIST2H2AC,FOSB,HIST1H2AB,HIST1H2AC,HIST1H2AG,HIST1H2AD,HIST1H2BB,HIST1H2BD,NRAS,HIST1H2AH,HIST1H2AJ
Nucleosome assembly(R)	45	10	0	<1.429E-04	HIST1H2BN,HIST1H2BJ,HIST1H4A,HIST2H2AC,HIST1H2AB,HIST1H2AC,HIST1H2AD,HIST1H2BB,HIST1H2BD,HIST1H2AJ
Senescence-Associated Secretory Phenotype (SASP)(R)	75	13	0	<1.667E-04	HIST1H2BN,HIST1H2BJ,FZR1,HIST1H4A,HIST2H2AC,UBE2C,HIST1H2AB,HIST1H2AC,HIST1H2AD,HIST1H2BB,HIST1H2BD,RPS6KA2,HIST1H2AJ
Meiotic Synapsis(R)	57	12	0	<2.000E-04	HIST1H2BN,HIST1H2BJ,STAG3,HIST1H4A,SMC1A,HIST2H2AC,HIST1H2AB,HIST1H2AC,HIST1H2AD,HIST1H2BB,HIST1H2BD,HIST1H2AJ
Meiotic Recombination(R)	54	12	0	<2.500E-04	HIST1H2BN,HIST1H2BJ,MRE11A,HIST1H4A,MLH1,HIST2H2AC,HIST1H2AB,HIST1H2AC,HIST1H2AD,HIST1H2BB,HIST1H2BD,HIST1H2AJ
Chromatin modifying enzymes(R)	105	16	0	<3.333E-04	MBIP,HIST1H2BN,HIST1H2BJ,ZZZ3,HIST1H4A,TAF9,HIST2H2AB,HIST2H2AC,HIST1H2AB,HIST1H2AC,HIST1H2AG,HIST1H2AD,HIST1H2BB,HIST1H2BD,HIST1H2AH,HIST1H2AJ
Telomere Maintenance(R)	59	13	0	<5.000E-04	HIST1H2BN,HIST1H2BJ,LIG1,HIST1H4A,HIST2H2AC,HIST1H2AB,HIST1H2AC,HIST1H2AD,POLA1,POLA2,HIST1H2BB,HIST1H2BD,HIST1H2AJ
DNA Damage/Telomere Stress Induced Senescence(R)	58	16	0	<1.000E-03	HIST1H2BN,HIST1H2BJ,MRE11A,HIST1H4A,HIST2H2AC,HIST1H1E,HIST1H1D,HIST1H1B,HIST1H1A,HMGA2,HIST1H2AB,HIST1H2AC,HIST1

					H2AD,HIST1H2BB,HIST1H2BD,HIST1H2AJ
Systemic lupus erythematosus(K)	135	13	0	<8.333E-05	HIST1H2BN,HIST1H2BJ,HIST1H4A,HIST2H2AB,HIST2H2AC,HIST1H2AB,HIST1H2AC,HIST1H2AG,HIST1H2AD,HIST1H2BB,HIST1H2BD,HIST1H2AH,HIST1H2AJ
Oxidative Stress Induced Senescence(R)	89	11	0	<9.091E-05	CBX6,HIST1H2BN,HIST1H2BJ,HIST1H4A,HIST2H2AC,HIST1H2AB,HIST1H2AC,HIST1H2AD,HIST1H2BB,HIST1H2BD,HIST1H2AJ
Direct p53 effectors(N)	133	10	0.0001	3.54E-03	EGFR,PIDD,HSPA1A,TAF9,ATF3,PLK3,MLH1,GADD45A,SNAI2,CCNB1
Biosynthesis of amino acids(K)	73	7	0.0002	7.21E-03	ASS1,ASL,PFKL,CTH,PHGDH,PSAT1,CBS
Cysteine and methionine metabolism(K)	34	5	0.0002	8.33E-03	DNMT3B,DNMT3A,TST,CTH,CBS
Cholesterol biosynthesis(R)	19	4	0.0003	8.94E-03	MVD,HMGCS1,FDPS,HSD17B7
g-protein signaling through tubby proteins(B)	10	3	0.0006	1.74E-02	EDN1,GNGT1,GNAQ
Metabolism of amino acids and derivatives(R)	147	9	0.0007	1.87E-02	ASS1,ASL,TST,GLS,CTH,ASNS,PHGDH,PSAT1,CBS
Dissolution of Fibrin Clot(R)	11	3	0.0008	2.14E-02	PLAT,PLAU,SERPINB2
Unfolded Protein Response(R)	74	6	0.0013	3.21E-02	ATF3,TPP1,ASNS,HSPA5,MBTPS2,HSP90B1
Estrogen signaling pathway(K)	100	7	0.0012	3.23E-02	CREB3L4,EGFR,HSPA1A,GNAQ,HSPA8,NRAS,HSP90B1
Apoptotic execution phase(R)	52	5	0.0016	3.70E-02	CLSPN,HIST1H1E,HIST1H1D,HIST1H1B,HIST1H1A
Alanine, aspartate and glutamate metabolism(K)	32	4	0.0019	4.09E-02	ASS1,ASL,GLS,ASNS

(B) BioCarta, (K) KEGG Pathway, (N) NCI - Nature Curated Data, (P) pantherdb, (R) Reactome
