# Supplementary material for: Integrative analysis of the microRNA-mRNA response to radiochemotherapy in primary head and neck squamous cell carcinoma cells
Source: BMC Genomics. 2015 Sep 2;16(1):654. doi: 10.1186/s12864-015-1865-x (PMC4557600; doi:10.1186/s12864-015-1865-x)
Supplement: Additional file 10: — Potential target genes in HN1957 for miRNAs responding to therapy in HNSCC patients. (PDF 72 kb) [file 12864_2015_1865_MOESM10_ESM.pdf]

Additional file 10 Potential target genes in HN1957 for miRNAs responding to therapy in HNSCC patients (correlation value ≤ -0.5)

miRNA	Gene	Correlation value
miR-106b-5p	ABTB2	-0.71
miR-106b-5p	ADH5	-0.66
miR-106b-5p	ANKFY1	-0.50
miR-106b-5p	APLP2	-0.63
miR-106b-5p	APP	-0.66
miR-106b-5p	BAMBI	-0.85
miR-106b-5p	BRWD1	-0.75
miR-106b-5p	C1orf43	-0.61
miR-106b-5p	CNOT7	-0.63
miR-106b-5p	CWF19L1	-0.72
miR-106b-5p	DDX23	-0.55
miR-106b-5p	DGKD	-0.82
miR-106b-5p	EEF1A1	-0.76
miR-106b-5p	FAM199X	-0.55
miR-106b-5p	FAM91A1	-0.77
miR-106b-5p	FBXO22	-0.54
miR-106b-5p	FBXW2	-0.66
miR-106b-5p	FNBP1	-0.73
miR-106b-5p	GOLT1B	-0.67
miR-106b-5p	GON4L	-0.76
miR-106b-5p	KAT7	-0.77
miR-106b-5p	MAT2A	-0.58
miR-106b-5p	MMADHC	-0.56
miR-106b-5p	PDSS1	-0.61
miR-106b-5p	PPIA	-0.78
miR-106b-5p	PPM1B	-0.64
miR-106b-5p	PTEN	-0.69
miR-106b-5p	PURA	-0.71
miR-106b-5p	RAD23B	-0.64
miR-106b-5p	RHOBTB2	-0.70
miR-106b-5p	RNPS1	-0.82
miR-106b-5p	RPL18A	-0.81
miR-106b-5p	RPP30	-0.79
miR-106b-5p	SHISA2	-0.56
miR-106b-5p	SMEK2	-0.75
miR-106b-5p	SMG7	-0.75
miR-106b-5p	THADA	-0.57

miR-106b-5p	TTPAL	-0.62
miR-106b-5p	UQCRQ	-0.59
miR-106b-5p	VEGFA	-0.68
miR-106b-5p	WDR33	-0.79
miR-106b-5p	YTHDF2	-0.53
miR-106b-5p	ZNF562	-0.72
miR-106b-5p	ZNF587	-0.63
miR-106b-5p	ZNF598	-0.78
miR-106b-5p	ZNF777	-0.66
miR-21-5p	AKT2	-0.58
miR-21-5p	APAF1	-0.59
miR-21-5p	BASP1	-0.64
miR-21-5p	DOCK5	-0.52
miR-21-5p	E2F1	-0.59
miR-21-5p	ISCU	-0.92
miR-21-5p	MARCKS	-0.51
miR-21-5p	MSH6	-0.56
miR-21-5p	NCAPG	-0.81
miR-21-5p	NFIB	-0.66
miR-21-5p	PCBP1	-0.58
miR-21-5p	PDCD4	-0.60
miR-21-5p	PPARA	-0.70
miR-21-5p	RECK	-0.63
miR-21-5p	SMARCA4	-0.54
miR-21-5p	SP1	-0.63
miR-21-5p	TMEM147	-0.56
miR-21-5p	TOPORS	-0.52
miR-425-5p	BAZ2A	-0.59
miR-425-5p	DPYSL2	-0.81
miR-425-5p	FOXK2	-0.67
miR-425-5p	HSP90AA1	-0.81
miR-425-5p	INPP5E	-0.79
miR-425-5p	NADK	-0.67
miR-425-5p	PDZD8	-0.53
miR-425-5p	QKI	-0.57
miR-425-5p	RRM2	-0.75
miR-425-5p	RUFY2	-0.65
miR-425-5p	SMARCD1	-0.57
miR-425-5p	THOP1	-0.67
miR-425-5p	ZNF148	-0.77
miR-93-5p	AP2A2	-0.72

miR-93-5p	APBB2	-0.54
miR-93-5p	APLP2	-0.71
miR-93-5p	ARHGAP32	-0.67
miR-93-5p	ATP5B	-0.57
miR-93-5p	CAP1	-0.73
miR-93-5p	CDC16	-0.78
miR-93-5p	CLN8	-0.61
miR-93-5p	DYRK2	-0.54
miR-93-5p	EEF1A1	-0.88
miR-93-5p	EIF4G2	-0.70
miR-93-5p	ELAC2	-0.52
miR-93-5p	ELAVL1	-0.81
miR-93-5p	EPHA4	-0.67
miR-93-5p	FAM3C	-0.56
miR-93-5p	GAPDH	-0.69
miR-93-5p	H1F0	-0.58
miR-93-5p	HK1	-0.69
miR-93-5p	HPS1	-0.73
miR-93-5p	IBA57	-0.76
miR-93-5p	IGF2	-0.76
miR-93-5p	IPO5	-0.88
miR-93-5p	JUN	-0.79
miR-93-5p	KAT2B	-0.57
miR-93-5p	LATS2	-0.83
miR-93-5p	LOXL1	-0.81
miR-93-5p	MAP3K13	-0.62
miR-93-5p	MED21	-0.64
miR-93-5p	NOB1	-0.75
miR-93-5p	NPEPPS	-0.57
miR-93-5p	NPM1	-0.66
miR-93-5p	NUP205	-0.74
miR-93-5p	PI4KA	-0.64
miR-93-5p	PPAN	-0.64
miR-93-5p	PPTC7	-0.77
miR-93-5p	PTEN	-0.68
miR-93-5p	PURA	-0.74
miR-93-5p	RAB8B	-0.61
miR-93-5p	RBM23	-0.53
miR-93-5p	RFC3	-0.77
miR-93-5p	RPL30	-0.61
miR-93-5p	SAMD4B	-0.67

miR-93-5p	SH3BP4	-0.62
miR-93-5p	SMG7	-0.69
miR-93-5p	SPATA2	-0.70
miR-93-5p	TAF8	-0.78
miR-93-5p	TRAK1	-0.59
miR-93-5p	TRIM8	-0.64
miR-93-5p	UBAP2L	-0.61
miR-93-5p	UBN1	-0.57
miR-93-5p	UPF1	-0.57
miR-93-5p	VAT1	-0.55
miR-93-5p	VEGFA	-0.53
miR-93-5p	WAC	-0.60
miR-93-5p	YWHAQ	-0.69
miR-93-5p	ZIC2	-0.53
miR-93-5p	ZNFX1	-0.68
