# Supplementary material for: Integrative analysis of the microRNA-mRNA response to radiochemotherapy in primary head and neck squamous cell carcinoma cells
Source: BMC Genomics. 2015 Sep 2;16(1):654. doi: 10.1186/s12864-015-1865-x (PMC4557600; doi:10.1186/s12864-015-1865-x)
Supplement: Additional file 11: — Potential target genes in HN2092 for miRNAs responding to therapy in HNSCC patients. (PDF 51 kb) [file 12864_2015_1865_MOESM11_ESM.pdf]

Additional file 11 Potential target genes in HN2092 for miRNAs responding to therapy in HNSCC patients (correlation value ≤ -0.5)

miRNA	Gene	Correlation value
miR-106b-5p	BRWD1	-0.60
miR-106b-5p	C9orf40	-0.59
miR-106b-5p	CCND2	-0.53
miR-106b-5p	INSIG1	-0.54
miR-106b-5p	ITCH	-0.56
miR-106b-5p	MSH6	-0.50
miR-106b-5p	PLEKHM1	-0.73
miR-106b-5p	PSMA3	-0.52
miR-106b-5p	RBL2	-0.59
miR-106b-5p	RBM12B	-0.68
miR-106b-5p	RPL7	-0.58
miR-106b-5p	SLC9A1	-0.51
miR-106b-5p	STX12	-0.55
miR-106b-5p	THADA	-0.51
miR-106b-5p	UGP2	-0.62
miR-106b-5p	ZBTB7B	-0.54
miR-106b-5p	ZFYVE16	-0.63
miR-21-5p	LRRFIP1	-0.53
miR-21-5p	PPARA	-0.51
miR-425-5p	NPNT	-0.53
miR-93-5p	AP2A2	-0.87
miR-93-5p	ARL9	-0.54
miR-93-5p	ATP5B	-0.62
miR-93-5p	BAG2	-0.51
miR-93-5p	BIRC5	-0.85
miR-93-5p	CBX3	-0.66
miR-93-5p	CCDC88C	-0.84
miR-93-5p	CCT8	-0.79
miR-93-5p	EIF4G2	-0.74
miR-93-5p	FAM3C	-0.81
miR-93-5p	FAM57A	-0.67
miR-93-5p	GAPDH	-0.81
miR-93-5p	HK1	-0.66
miR-93-5p	HPS1	-0.62
miR-93-5p	IBA57	-0.90
miR-93-5p	IGF2	-0.55
miR-93-5p	IPO5	-0.87

miR-93-5p	JUN	-0.79
