# Supplementary material for: Integrative analysis of the microRNA-mRNA response to radiochemotherapy in primary head and neck squamous cell carcinoma cells
Source: BMC Genomics. 2015 Sep 2;16(1):654. doi: 10.1186/s12864-015-1865-x (PMC4557600; doi:10.1186/s12864-015-1865-x)
Supplement: Additional file 12: — Primer sequences for TP53 and EGFR sequencing. (PDF 42 kb) [file 12864_2015_1865_MOESM12_ESM.pdf]

Additional file 12 Primer sequences for *TP53* and *EGFR* sequencing

Gene (region)	PCR Primer 5' → 3'	Sequencing Primer 5' → 3'
TP53 (exons 3-10)	ATGGAGGAGCCGCAGTCAG<br>TCAGTCTGAGTCAGGCCCTTCT	ATGGAGGAGCCGCAGTCAG<br>TCAGTCTGAGTCAGGCCCTTCT
EGFR (exons 2-16)	ATGCGACCCTCCGGGAC<br>CACCACCAGCAGCAAGAGG	ATGCGACCCTCCGGGAC<br>CACCACCAGCAGCAAGAGG
EGFR (exons 9-27)	CCGACAGCTATGAGATGGAGGA<br>TCATGCTCCAATAAATTCACTGCT	CCGACAGCTATGAGATGGAGGA<br>CCTCTTGCTGCTGGTGGTG<br>TCATGCTCCAATAAATTCACTGCT
EGFR (exons 9-14)	CCTCTTGCTGCTGGTGGTG<br>ATCGATGGTACATATGGGTGGC	CCTCTTGCTGCTGGTGGTG<br>ATCGATGGTACATATGGGTGGC
